# Interaction between Two Timing MicroRNAs Controls Trichome Distribution in *Arabidopsis*

**DOI:** 10.1371/journal.pgen.1004266

**Published:** 2014-04-03

**Authors:** Xue-Yi Xue, Bo Zhao, Lu-Men Chao, Dian-Yang Chen, Wen-Rui Cui, Ying-Bo Mao, Ling-Jian Wang, Xiao-Ya Chen

**Affiliations:** 1 National Key Laboratory of Plant Molecular Genetics and National Plant Gene Research Center, Institute of Plant Physiology and Ecology, Shanghai Institutes for Biological Sciences, Chinese Academy of Sciences, Shanghai, China; 2 University of Chinese Academy of Sciences, Beijing, China; 3 Plant Science Research Center, Shanghai Chenshan Botanical Garden, Shanghai, China; University of California Riverside, United States of America

## Abstract

The miR156-targeted *SQUAMOSA PROMOTER BINDING PROTEIN LIKE* (*SPL*) transcription factors function as an endogenous age cue in regulating plant phase transition and phase-dependent morphogenesis, but the control of SPL output remains poorly understood. In *Arabidopsis thaliana* the spatial pattern of trichome is a hallmark of phase transition and governed by SPLs. Here, by dissecting the regulatory network controlling trichome formation on stem, we show that the miR171-targeted *LOST MERISTEMS 1* (*LOM1*), *LOM2* and *LOM3*, encoding GRAS family members previously known to maintain meristem cell polarity, are involved in regulating the SPL activity. Reduced LOM abundance by overexpression of miR171 led to decreased trichome density on stems and floral organs, and conversely, constitutive expression of the miR171-resistant *LOM* (*rLOM*) genes promoted trichome production, indicating that LOMs enhance trichome initiation at reproductive stage. Genetic analysis demonstrated LOMs shaping trichome distribution is dependent on SPLs, which positively regulate trichome repressor genes *TRICHOMELESS 1* (*TCL1*) and *TRIPTYCHON* (*TRY*). Physical interaction between the N-terminus of LOMs and SPLs underpins the repression of SPL activity. Importantly, other growth and developmental events, such as flowering, are also modulated by LOM-SPL interaction, indicating a broad effect of the LOM-SPL interplay. Furthermore, we provide evidence that *MIR171* gene expression is regulated by its targeted LOMs, forming a homeostatic feedback loop. Our data uncover an antagonistic interplay between the two timing miRNAs in controlling plant growth, phase transition and morphogenesis through direct interaction of their targets.

## Author Summary

MicroRNAs are important aging regulators in many organisms. In *Arabidopsis* the miR156-targeted *SQUAMOSA PROMOTER BINDING PROTEIN LIKE* (*SPL*) transcription factors play important roles as an endogenous age cue in programming phase transition and phase-dependent morphogenesis, including trichome patterning. However, how the timely increasing SPL output is modulated remains elusive. By dissecting the regulatory network controlling trichome formation on stem, we show that a group of GRAS family members, LOST MERISTEMS 1 (LOM1), LOM2 and LOM3, targeted by timing miR171, function in modulating the SPL activity through direct protein-protein interaction. LOMs promote trichome formation through attenuating the SPL (such as SPL9) activity of trichome repression. The LOM-SPL interaction affects many aspects of plant growth and development, including flowering, aging and chlorophyll biosynthesis. Interestingly, *MIR171A* gene expression is regulated by its own targets (LOMs), forming a feedback loop to program plant life. Our study establishes an age-dependent regulatory network composed of two timing miRNAs which act oppositely through direct interaction of their target proteins.

## Introduction

MicroRNA (miRNA) was first identified as the regulator of the juvenile-to-adult transition in *Caenorhabditis elegans* [1], and a similar function was later assigned to plant miRNA: miR156 and its target *SQUAMOSA PROMOTER BINDING PROTEIN LIKE* (*SPL*) genes define an endogenous aging and flowering pathway [2], [3]. The miR156 overexpression delays phase transition and startup of flowering. Similarly, accumulation of SPLs accelerates aging [2]–[4]. In *Arabidopsis* there are 17 *SPL* genes, 11 of which contain a miR156 target site. The miR156-targeted SPLs can be divided into four clades based on their protein structures, and the clades composed of SPL3/4/5 and SPL9/15 both promote phase transition [2]–[4]. Acting downstream of miR156-targeted SPLs, miR172 also plays roles in developmental timing of *Arabidopsis* [3]. In addition, there are other transcription regulators that affect SPL activities, such as DELLAs, the negative regulator of gibberellin signaling pathway, interact with SPLs to control flowering [5].

Trichomes are specialized epidermal cells, acting as barriers to protect plants from herbivores, UV irradiation, and excessive transpiration. In *Arabidopsis*, trichome distribution is spatially and temporally regulated, and the distribution pattern serves as a trait to distinguish between juvenile and adult leaves [6], [7]. During the early vegetative phase, trichomes are evenly distributed on the adaxial side of rosette leaves. Plants start transition from juvenile to adult phase when trichomes initiate on leaf abaxial side [7]. After entering into the reproductive stage, the number of trichomes is gradually reduced along the inflorescence stems. Floral organs are nearly glabrous except for a few trichomes on the abaxial surface of sepals. Genetic screens of *Arabidopsis* mutants have identified sets of regulators governing trichome formation. In brief, the trichome initiation complex of *Arabidopsis* comprises a WD40 protein TRANSPARENT TESTA GLABRA1 (TTG1), an R2R3 MYB protein GLABRA1 (GL1), and a basic helix-loop-helix transcription factor GL3 or its homolog, ENHANCER of GL3 (EGL3) [8]–[10]. This ternary complex initiates trichome cell development by activating *GL2*, which encodes a homeodomain leucine zipper transcription factor [11], [12]. A group of single-repeat R3 MYB factors, including TRIPTYCHON (TRY) [13], CAPRICE (CPC) [14], [15], ENHANCER OF TRY AND CPC1 (ETC1) [16], ETC2 [17], ETC3 [18], TRICHOMELESS1 (TCL1) [19] and TCL2 [20], [21], redundantly suppress trichome initiation by competing with GL1 for the binding site of GL3 to prevent the active complex formation [12]. In addition to these specific negative factors, hormone signaling components also affect the trichome regulatory complex [22].

We previously reported that the miR156-targeted SPLs temporally repress trichome distribution on stem and inflorescence through activating *TCL1* and *TRY* [23]. Plants overexpressing miR156 developed ectopic trichomes on the stem and floral organs, whereas plants with elevated SPLs produced fewer trichomes after bolting [23]. Since miR156-SPLs define a major endogenous age cue, this provides a straightforward mechanism that connects plant phase transition with trichome development. Due to the easiness of observation, trichome formation on stem is an ideal system to investigate the interaction of miR156-SPL module with other developmental or environmental signaling pathways.

LOST MERISTEMS (LOMs), also known as AtHAMs because the first functionally characterized member of this subclade is HAIRY MERISTEM (HAM) from *Petunia hybrid* [24], are transcription factors belonging to the GRAS-domain containing family. There are three LOM genes in *Arabidopsis*, *LOM1*/*2*/*3* (also known as *AtHAM1*/2/3 [25] or *SCL6-2*/*3*/*4* [26]), which are targets of miR171 [27], [28]. Like other GRAS members, LOMs contain a highly conserved GRAS domain on C-terminus and a variable N-terminus. LOM1 and LOM2 are ∼65% identical at amino acid sequence level, and LOM3 has a shorter N-terminal domain. Previous studies revealed that LOMs function in diverse processes such as meristem maintenance, shoot branching, chlorophyll biosynthesis and root growth [25]–[27], [29]. Here, we report that miR171-targeted LOMs functionally interfere with selected SPLs through protein-protein interaction.

## Results

### miR171-LOM regulates trichome distribution

**Figure 1:**
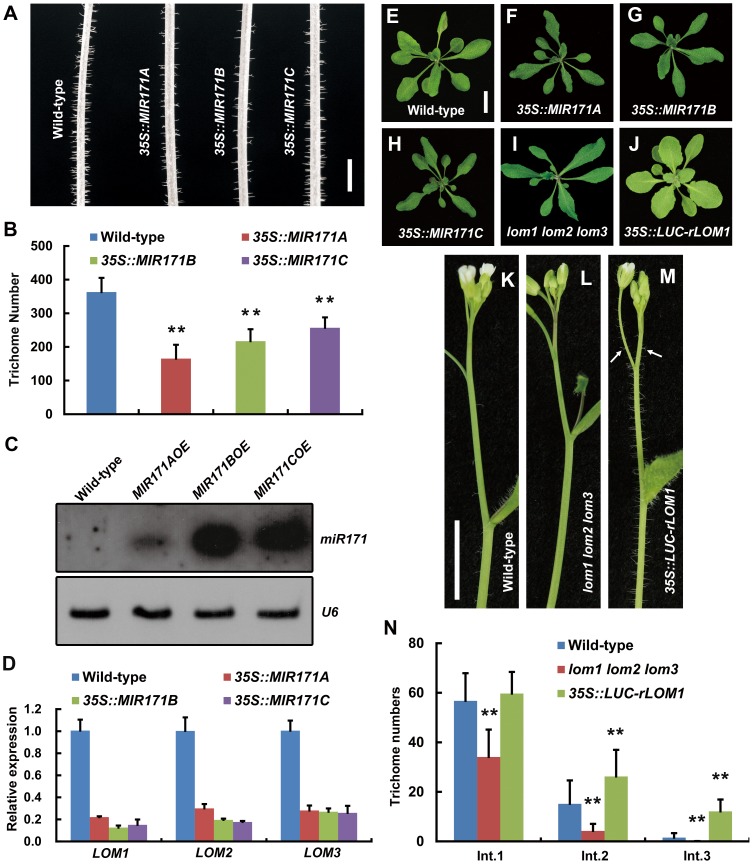
MiR171-LOMs regulate trichome initiation. (A) Trichome distribution on main stem of wild-type (Col-0) and *MIR171OE* (*35S::MIR171A*/*B*/*C*) plants; *MIR171* overexpression decreased trichome density. Scale bar represents 5 mm. (B) Overexpression of *MIR171* reduced the total number of trichomes on stems. Trichome numbers on main stems (0 to 5 cm from bottom) were counted and data were given as mean s.d. (*n* = 18) and analyzed by *t* test. **P<0.01. (C) RNA blots showing high accumulation of mature miR171 in 10-day-old *MIR171OE* plants. *U6* was used as an internal reference. (D) qRT-PCR showing repressed expression of *LOM1*, *LOM2* and *LOM3* in *MIR171OE* plants. Expression level in the wild-type was set to 1. Errors bars indicate s.d. (n = 3). Three biological replicates were analyzed, with consistent results. (E–J) View of 15-day-old plants of indicated genotypes. Overexpression of *MIR171* resulted in dark-green and narrower leaves. *MIR171OE* transgenic plants showed similar phenotypes as *lomt* (*lom1 lom2 lom3*) mutant (I). On the contrary, *35S::LUC-rLOM1* transgenic plants (J) showed yellow-green leaves. Scale bar in (E) represents 1 cm for (E–J). (K–M) Spatial distribution of trichomes on main stem of wild-type, *lomt* and *35S::LUC-rLOM1* plants. Arrows in (M) indicate the ectopic trichomes on the main inflorescence stems and pedicels. Scale bar in (K) represents 1 cm for (K–M). (N) Trichome density on main stem of wild-type, *lomt* and *35S::LUC-rLOM1* plants. The Int.1 to Int.3 represents the main stem internodes from bottom to top. The *y* axis indicates trichome number per centimeter of each internode. Data are given as mean s.d. (*n*>16) and analyzed by *t* test. **P<0.01.

In *Arabidopsis* genome, there are four miR171 coding genes, *MIR171A*, *B*, *C* and *MIR170*. We found that overexpression (OE) of *MIR171A*/*B*/*C* under the *35S* promoter reduced trichomes on stem very significantly (Figure 1A and 1B), which was not reported previously. To see if the reduction of trichomes was caused by decreased level of LOMs due to miR171 accumulation, we first checked the abundance of mature miR171 and the transcript levels of *LOMs* in these transgenic plants. Indeed, miR171 was over-accumulated (Figure 1C) and *LOM* expression declined drastically (Figure 1D). In addition, miR171 overexpression resulted in phenotypic changes similar to those of *lom1 lom2 lom3* triple mutant (termed *lomt* hereinafter) as reported [26]: the narrower rosette leaves and the higher chlorophyll content (Figure 1E–1I). We then analyzed the effects of LOM overaccumulation by examining the *35S::LUC-rLOM1* plants, in which a miRNA-resistant *LOM1* (*rLOM1*) was fused to firefly luciferase (LUC) gene and expressed constitutively. In addition to yellow-green leaves (Figure 1J) as reported [26], the *35S::LUC-rLOM1* plants produced supernumerary trichomes on stems, inflorescences and pedicels compared to the wild-type and *lomt* mutant plants (Figure 1K–1N), indicating that LOM1 accumulation induces ectopic trichomes after bolting.

**Figure 2:**
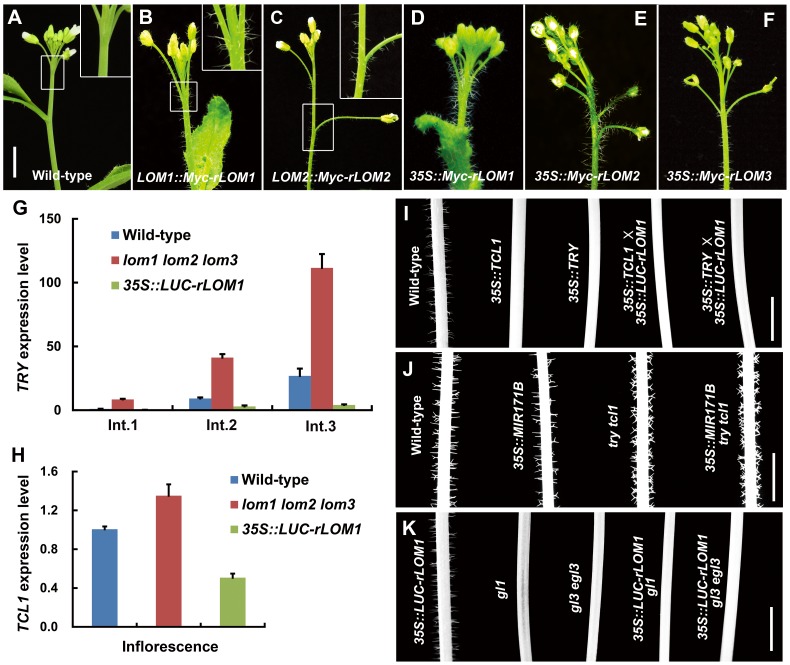
Elevated LOMs trigger ectopic trichomes and down-regulate *TRY* and *TCL1*. (A–F) Trichomes on main stems and inflorescences of different plants as indicated. The insertion panel in the top right corner in (A–C) represents magnified image of the area marked by square; note that ectopic trichomes appeared on inflorescence stems and pedicels in (B) and (C). Scale bar shared in (A–F), 1 cm. (G) Relative expression level of the trichome repressor gene *TRY* in main stem of wild-type, *lomt* and *LUC-rLOM1OE* plants. The Int.1 to Int.3 represent the stem internodes from bottom to top. Expression level in Int.1 of wild-type was set to 1. Error bars indicate s.d. (n = 3). (H) Relative expression level of *TCL1* in inflorescences of wild-type, *lomt* and *LUC-rLOM1OE* plants. Errors bars indicate s.d. (n = 3). For (G) and (H) three biological replicates were analyzed with similar results. (I–K) Trichome distribution on main stems (Int.1) of different genetic background plants as indicated. Note that *TCL1* and *TRY* are epistatic to *LOM1*, as *LUC-rLOM1OE* did not induce trichome production in *TCL1OE* and *TRYOE* backgrounds (I). *MIR171BOE* did not reduce trichome density in *try tcl1* double mutant (J). *35S::LUC-rLOM1* failed to rescue the glabrous phenotype of *gl1* and *gl3 egl3* mutants (K). Scale bars in (I–K) represent 1 cm.

To further examine the role of LOMs in trichome production, we analyzed transgenic *Arabidopsis* expressing Myc-tagged *rLOM1*, *2* and *3*, under the *35S* or their native promoters, respectively. *LOM1::Myc-rLOM1* and *LOM2::Myc-rLOM2* plants produced supernumerary or ectopic trichomes on stems, inflorescences, and even on pedicels that are glabrous in wild-type plants (Figure 2A–2C); and *35S::Myc-rLOM1*/*2*/*3* plants exhibited a similar phenotype as that of *35S::LUC-rLOM1* with higher but varied degrees of trichome enrichment (Figure 2D–2F). To exclude the effect of Myc tag on LOM activities, we generated *35S::rLOM1* transgenic *Arabidopsis*, which exhibited the same phenotypic change as *35S::Myc-rLOM1* and *35S::LUC-rLOM1*. Clearly, the three miR171-regulated LOMs have the ability to promote trichome initiation at flowering stage.

### Stem trichome repressors act at downstream of miR171-LOMs

Based on the *Arabidopsis* trichome model [12], ectopic trichomes could be triggered by either the increase of trichome promoting factors or the reduction of single-repeat R3 MYB repressors. For example, gibberellin and cytokinin promote trichome formation by activating the expression of C2H2 transcription factor genes *GIS*, *GIS2* and *ZFP8*, which successively promote the transcription of *GL1* and *GL3* [30]–[32]. However, analysis of the *lomt* and the *35S::LUC-rLOM1* plants by quantitative RT-PCR (qRT-PCR) did not show an evident change of transcript levels of *GIS*, *GIS2*, *ZFP8* and *GL3* (Figure S1A). We then examined the expression of the R3 MYB repressor genes and found that transcript changes of *CPC*, *ETC1* and *TCL2* were uncoupled from trichome production in the *lomt* and the *35S::LUC-rLOM1* plants (Figure S1B and S1C), whereas the mRNA levels of *TRY* and *TCL1* matched the phenotypes (Figures 2G and 2H). Indeed, among these single MYB-domain factors, TRY and TCL1 are the major negative regulators of stem and inflorescence trichomes [13], [19], [23]. *TRY* expression in the main stems was up-regulated in *lomt* and repressed in *35S::LUC-rLOM1* plants (Figure 2G). And similarly, *TCL1* expression was also down-regulated in *35S::LUC-rLOM1* inflorescences (Figure 2H), consistent with the ectopic trichomes on inflorescence stems and pedicels. These data indicate that transcriptional repression of *TRY* and *TCL1* occurred in LOM-overexpressors that produced ectopic trichomes.

To dissect genetic interaction between *LOMs* and the trichome repressor genes, we crossed *35S::LUC-rLOM1* to *35S::TRY* and *35S::TCL1* plants, respectively. Overexpression of LOM1 failed to induce trichome production as both hybrid progeny exhibited the glabrous phenotype like *35S::TRY* or *35S::TCL1* (Figure 2I). On the other hand, down-regulation of *LOMs* in *try tcl1* double mutant by *MIR171B* overexpression did not reduce trichome production as it worked in wild-type background (Figure 2J). These data indicate that miR171-LOMs act upstream of *TRY* and *TCL1* in promoting trichome formation. We also introduced *35S::LUC-rLOM1* into *gl1* and *gl3 egl3* backgrounds respectively, and the resultant plants barely developed trichomes (Figure 2K), demonstrating that, whether promoted by LOMs or not, trichome initiation in *Arabidopsis* requires the GL1-GL3/EGL3-TTG1 ternary complex.

### LOMs attenuate SPL activities of trichome repression

**Figure 3:**
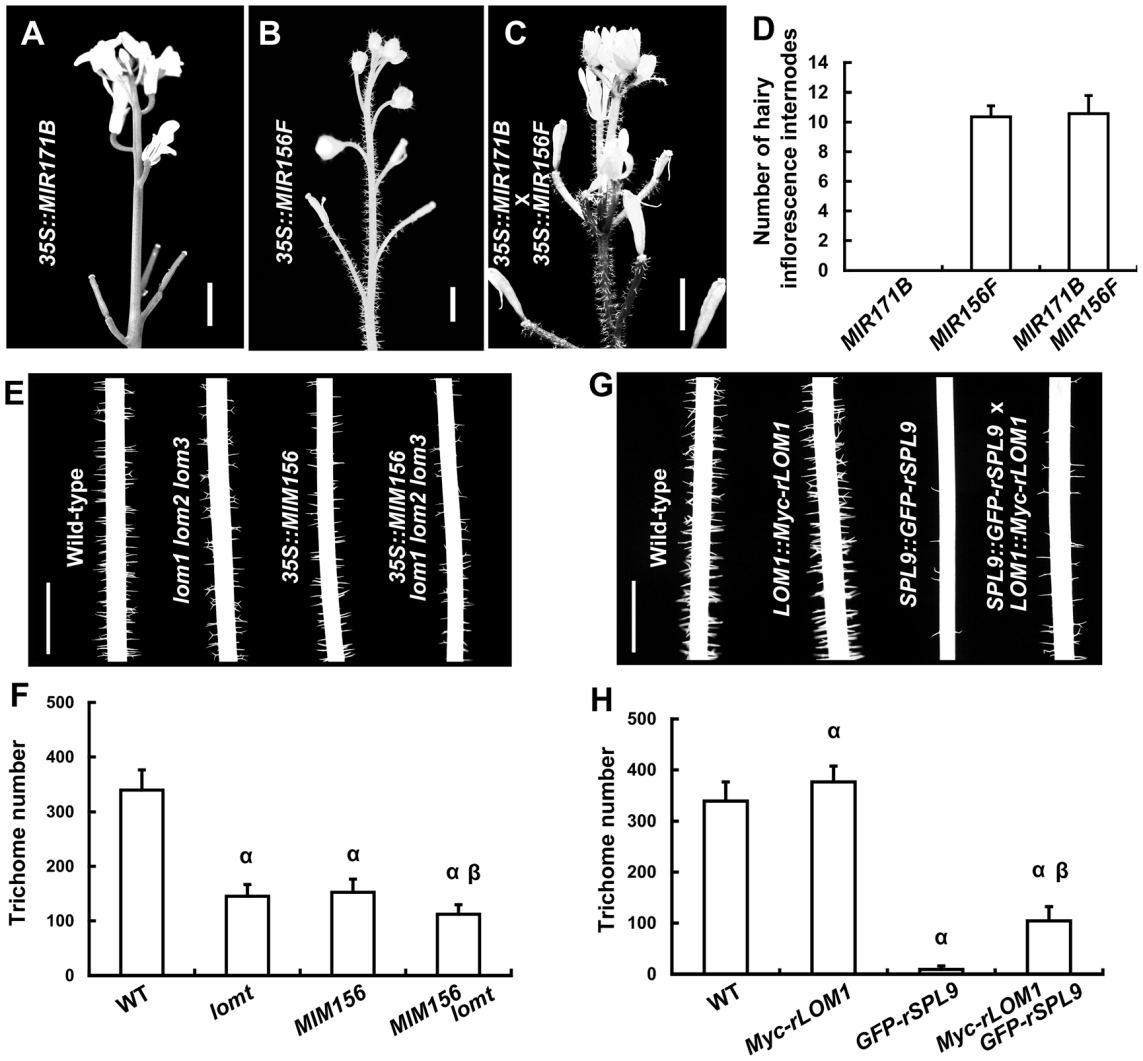
LOMs and SPLs regulate trichome formation antagonistically. (A–C) Trichome distribution on stems and inflorescences of *35S::MIR171B* (A), *35S::MIR156F* (B) and the progeny of *35S::MIR171B*×*35S::MIR156F* plants (C). Bars = 5 mm in (A–C). (D) Number of main inflorescence stem internodes with trichomes, counting from the first pedicel. Data are given as mean s.d. (*n* = 14). (E) Trichomes on stems (Int.1) of the indicated genotypes; note that trichome production was further reduced in *35S::MIM156 lomt* plants. (F) Trichome numbers of plants in (E). The *y* axis indicates trichome number on main stem (0 to 5 cm from the bottom). Data are given as mean s.d. (*n* = 20); α represents *P* values (*t*-test)<0.01 relative to wild-type, and β represents *P* values (*t*-test)<0.01 relative to *lomt* and *MIM156*, respectively. Note that the trichome number of *MIM156 lomt* was very significantly less than that in *MIM156* or *lomt* plants. (G) Trichomes on the stems (Int.1) of the indicated genotypes. *SPL9::GFP-rSPL9* transgenic plants were almost glabrous, and the *LOM1::Myc-rLOM1* plants produced more trichomes than the wild-type. (H) Trichome numbers (0 to 5 cm from the basal of main stems) of plants in (G). Data are given as mean s.d. (*n* = 20). α represents *P* values (*t*-test)<0.01 relative to wild-type, and β represents *P* values (*t*-test)<0.01 relative to *Myc-rLOM1* and *GFP-rSPL9*, respectively.

Our previous report showed that SPLs repress stem and inflorescence trichome production through activating *TCL1* and *TRY* gene expression [23]. Because up-regulation of *LOMs* (*35S::LUC-rLOM1*) and down-regulation of *SPLs* (*35S::MIR156F*) both reduced *TCL1* and *TRY* expression and triggered ectopic trichomes, we wondered if miR171-LOMs induced trichome formation through affecting the miR156-targeted SPLs. To test this hypothesis, we crossed *35S::MIR171B* to *35S::MIR156F* plants. Although *35S::MIR171B* repressed trichome formation on stem in wild-type plants, it did not change the ectopic trichome distribution induced by *35S::MIR156F* (Figure 3A–3D), suggesting a requirement of miR156-targeted SPLs in miR171-mediated trichome suppression. Correspondently, mutation of *LOM* genes caused a further trichome reduction in *35S::MIM156* plants (Figure 3E and 3F), suggesting an enhancement of SPL activity in the absence of LOMs. High level of LOM1 (*LOM1::Myc-rLOM1*) stimulated while high level of SPL9 (*SPL9::GFP-rSPL9*) repressed stem trichome formation (Figure 3G and 3H). The plants expressing both (*LOM1::Myc-rLOM1*×*SPL9::GFP-rSPL9*) showed an intermediate phenotype in terms of stem trichomes, though less than the wild-type (Figure 3G and 3H). Together, these data suggest an antagonistic effect of LOMs on the SPLs. Although the intermediate phenotype mentioned above, at this stage it does not rule out the possibility of independent effects of these proteins on common downstream targets, such as *TRY* and *TCL1*.

### LOMs interact with SPLs

**Figure 4:**
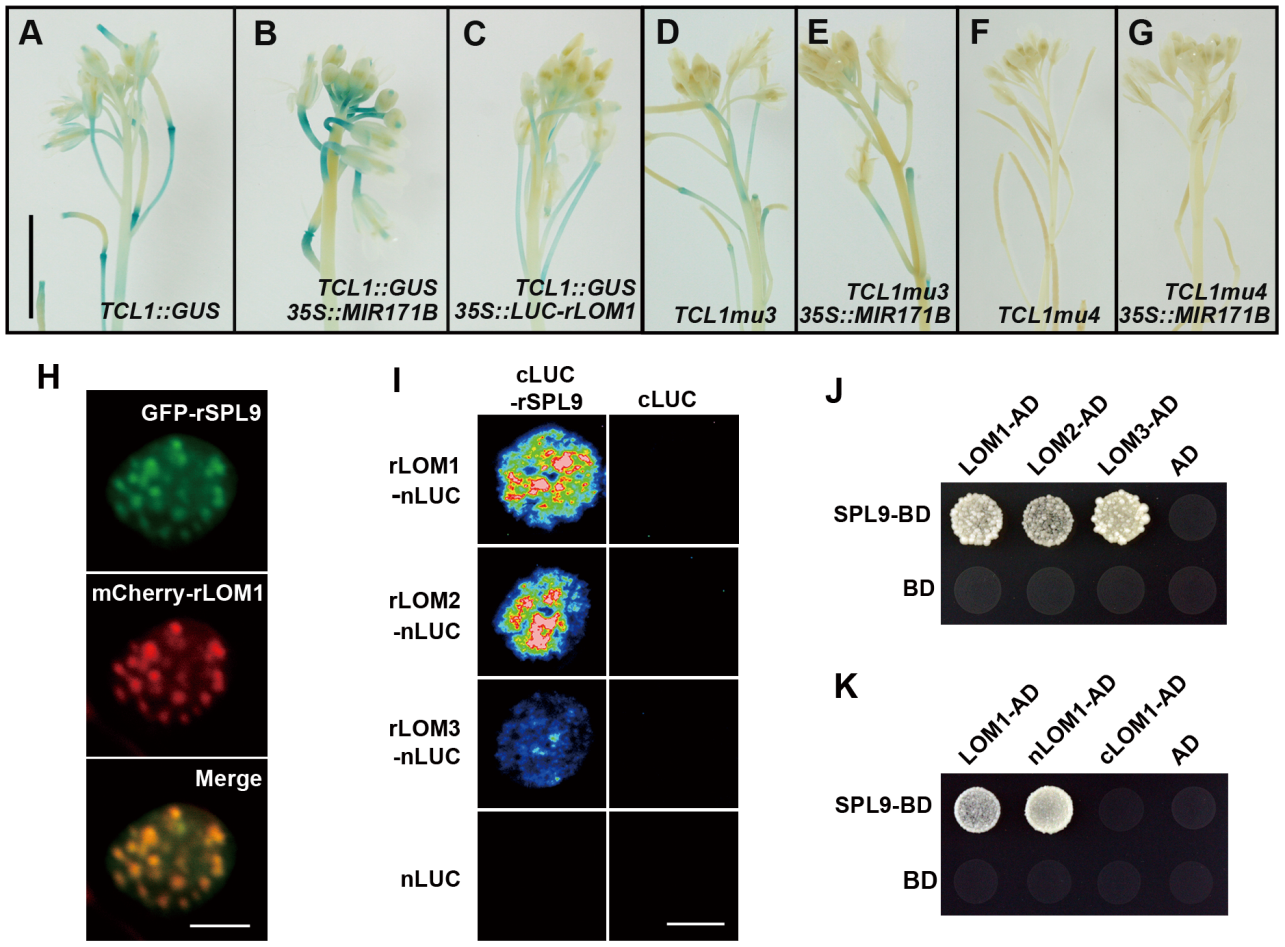
LOMs repress SPL function through direct protein-protein interaction. (A–C) *TCL1::GUS* reporter in indicated backgrounds. GUS activity was increased in *MIR171BOE* (B) and decreased in *LUC-rLOM1OE* (C) compared to wild-type (A) inflorescences. (D–G) The increase of GUS activity in *MIR171BOE* plants was abolished when the SPL binding sites in *TCL1* promoter was mutated (see [23]). *MIR171BOE* did not increase the activity of *TCL1mu3::GUS* (E) and *TCL1mu4::GUS* (G), compared to that shown in (D) and (F), respectively. Scale bar in (A) represents 1 cm for (A–G). (H) SPL9 and LOM1 were co-localized in nucleus. *35S::GFP-rSPL9* and *35S::mCherry-rLOM1* were transiently expressed in *N. benthamiana* leaves and the fluorescence merged in nucleus. Scale bar, 10 µm. (I) BiFC assay of the interaction between LOMs and SPL9. *rLOMs-nLUC* or *nLUC* was transiently co-expressed with *cLUC-rSPL9* or *cLUC* in *N. benthamiana* leaves; only the combination of *rLOMs-nLUC* and *cLUC-rSPL9* triggered the luciferase activity when luciferin was infiltrated. Scale bar, 1 cm. (J) Yeast two-hybrid assay of protein-protein interaction. SPL9-BD combined with LOM1/2/3-AD conferred yeast growth in -Leu-Trp-His medium. (K) SPL9 interacts with the N-terminal (1–250) of LOM1 (nLOM1) in yeast two-hybrid system.

In order to further elucidate the correlation between LOMs and SPLs in trichome regulation, we analyzed *TCL1* promoter activities in plants of different backgrounds. GUS activity conferred by *TCL1::GUS* was decreased in *35S::LUC-rLOM1* and increased in 35S::*MIR171B* plants (Figure 4A–4C), consistent with qRT-PCR results (Figure 2H). The increase of GUS activity in 35S::*MIR171B* was abolished when the mutant promoters of *TCL1mu3::GUS* and *TCL1mu4::GUS* (see [23]) were used, in which the SPL binding sites in *TCL1* promoter were disrupted (Figures 4D–4G). Clearly, the SPL binding motifs were involved in regulation of *TCL1* by LOMs, further supporting that LOMs modulate trichome formation via SPLs. *35S::LUC-rLOM1* and *35S::MIR156F* plants exhibited other phenotypic similarities in addition to ectopic trichome production, such as short plastochron, delayed flowering time, yellow-green leaves, enhanced shoot branching and limp stem (see [26] and Figure S2), suggesting that the antagonistic effects between LOMs and SPLs could be general rather than limited to trichome regulation. It was reported that DELLAs physically interact with SPLs and gibberellin promotes flowering partially through releasing this interaction [5]. Since both LOM and DELLA proteins belong to two close subclades of the GRAS family [33], we wondered if LOMs also interacted with SPLs. We first examined the subcellular localization of SPL9 and LOM1. *35S::GFP-rSPL9* and *35S::mCherry-rLOM1* were transiently expressed in *Nicotiana benthamiana* leaves, and the fusion proteins of GFP-SPL9 and mCherry-LOM1 were found co-localized in speckles in the nucleus (Figure 4H). We then performed a biomolecular fluorescence complementation (BiFC) assay by fusing SPL9 to the C-terminal half of LUC (cLUC-rSPL9) and LOMs to the N-terminal half (LOMs-nLUC). A strong LUC activity was detected in leaves co-infiltrated with the respective chimerical constructs (Figure 4I). The interaction between SPL9 and LOMs was further confirmed in a yeast two-hybrid assay (Figure 4J). Domain deletion revealed that the N-terminal domain of LOM1 was responsible for its interaction with SPL9 (Figure 4K). In addition, LOMs were able to bind to SPL2 (Figure S3A), which is remarkably also involved in trichome development [34]. Taken together, these results demonstrate that the miR171-targeted LOMs physically interact with the miR156-targeted SPL9 and SPL2 and may result in attenuation of the SPL function such as regulating trichome patterning. Based on the facts that miR156 and miR171 are conserved in plant kingdom and excessive miRNAs cause opposite phenotypes, the interaction between the two miRNA targets coordinate many developmental and morphogenesis events beyond trichome formation.

### LOMs repress SPL activities of gene regulation

**Figure 5:**
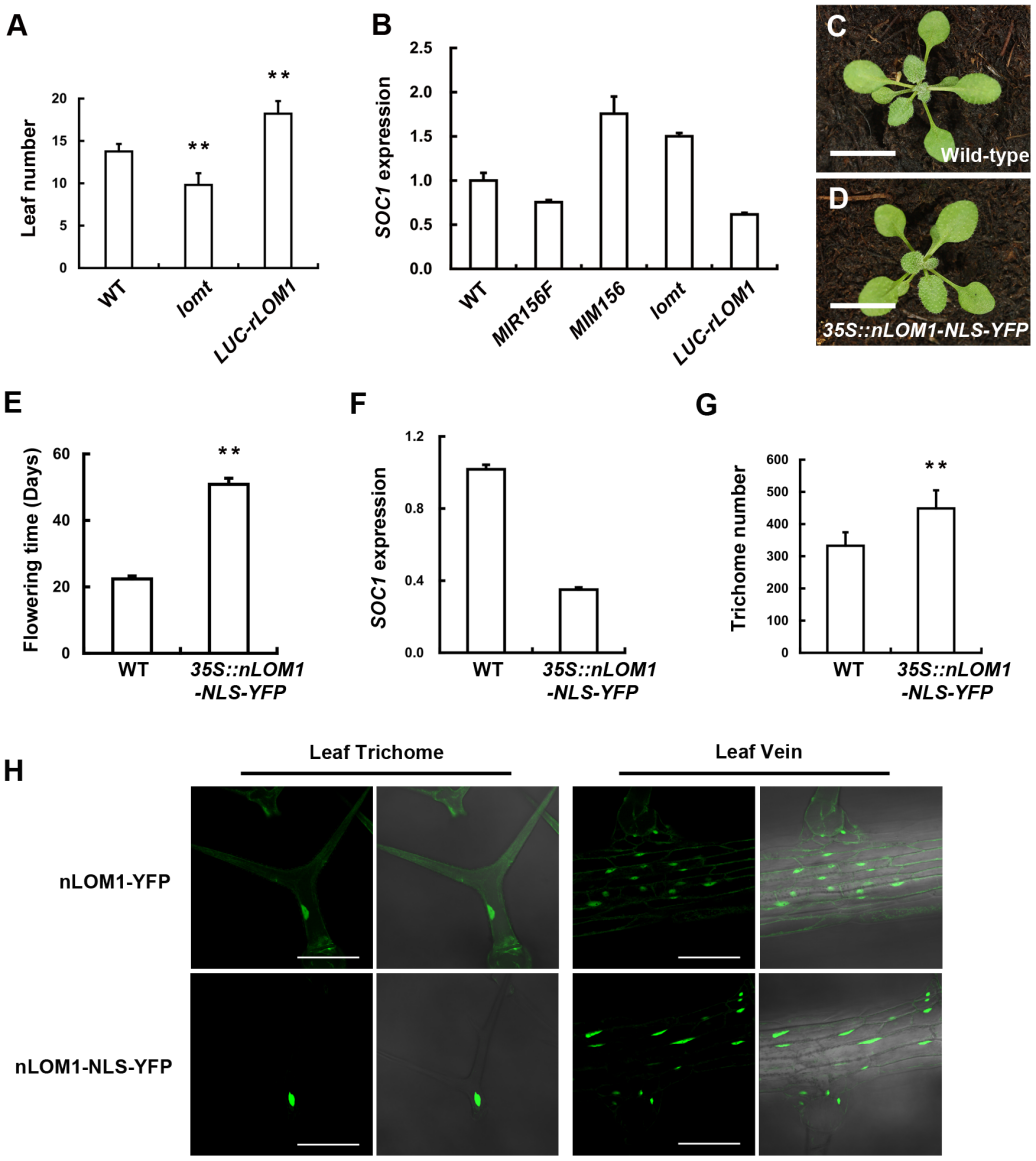
Overexpression of N-terminal of LOM1 in nucleus triggers a flowering delay. (A) The number of leaves at flowering of wild-type, *lomt* mutant and *LUC-rLOM1OE* plants under long-day condition. Data are given as mean s.d (n>61), and analyzed by *t* test. **P<0.01. (B) qRT-PCR analysis of expression of *SOC1* in 7-day-old long-day-grown seedlings. *SOC1* was repressed in *35S::MIR156F* and increased in *35S::MIM156* as reported [2]. (C and D) View of 10-day-old wild-type (C) and *35S::nLOM1-NLS-YFP* (D) plants. *35S::nLOM1-NLS-YFP* plants look normal. Scale bars, 1 cm. (E) *35S::nLOM1-NLS-YFP* delayed flowering very significantly under long-day condition. The number of days to flowering was counted when the floral buds were visible. Data are given as mean s.d. (*n* = 18) and analyzed by *t* test, **P<0.01. (F) Expression of *SOC1*. *SOC1* was down-regulated in *35S::nLOM1-NLS-YFP* plants, consistent with delayed flowering. (G) Trichome numbers of wild-type and *35S::nLOM1-NLS-YFP* plants. The *y* axis indicates the total trichome number on main stems. Data are given as mean s.d. (*n* = 32) and analyzed by *t* test, **P<0.01. (H) Subcellular localization of nLOM1-YFP and nLOM1-NLS-YFP in leaf trichome (left panel) and leaf vein (right panel). Scale bars, 100 µm.

To substantiate the potentially wide implication of LOM-SPL interaction, we examined the effect of LOMs on the flowering pathway. In long-day conditions, LOM1 overexpression delayed flowering time (Figure 5A and Figure S3B–S3E) and consistently down-regulated the expression of the MADS-box gene, *SUPPRESSOR OF OVEREXPRESSION OF CONSTANS 1* (*SOC1*), which was under the direct control of miR156-tageted SPLs [2]; by contrast, *SOC1* was up-regulated in *lomt* mutant along with earlier flowering (Figure 5A and 5B). As the N-terminal domain of LOM1 (nLOM1) was responsible for interaction with SPL9 (Figure 4K), we overexpressed nLOM1 fused to the nuclear localization signal (NLS). The *35S::nLOM1-NLS-YFP* plants showed normal development (Figures 5C and 5D) except for late flowering and more trichomes on stems (Figures 5E–5G). A drastic drop of the *SOC1* transcript levels (Figure 5F) was accompanied by severe delay of flowering (Figure 5E) in *35S::nLOM1-NLS-YFP* plants. It is the same to *rLOM1OE* plants that nLOM1-NLS-YFP triggered more stem trichomes (Figure 5G). However, remarkable flowering delay and ectopic trichomes were not observed in *35S::nLOM1-YFP* plants, in which the NLS was removed and the nLOM1-YFP signal was diffused in cytoplasm (Figure 5H). Because nLOM1 does not contain the DNA binding domain, these data further support that it is the LOM-SPL interaction that caused, or at least contributed to, the flowering delay and trichome increase.

We noted that, among the three LOMs, LOM3 has a shorter and more diversified N-terminal region (Figure S4). Interestingly, in comparison with *35S::Myc-rLOM1* and *35S::Myc-rLOM2* plants, overexpression of *rLOM3* only led to a mild increase of trichome production (Figure 2F) and less serious developmental abnormalities. This result again points to the importance of the N-terminal domains of LOM proteins.

### LOMs induce miR171 accumulation

**Figure 6:**
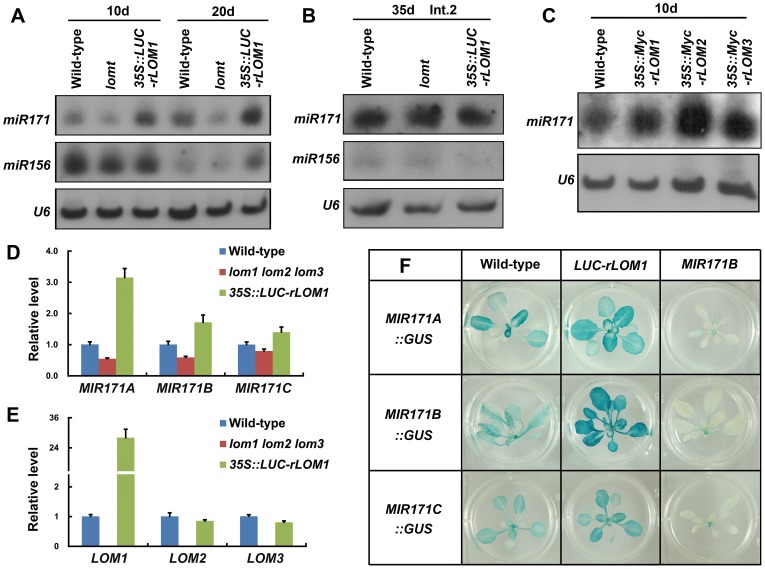
LOMs regulate *MIR171* expression. (A) RNA blot analysis of mature miR171 and miR156 in wild-type, *lomt* and *LUC-rLOM1OE* plants. Seedlings (10-day-old) and rosette leaves (20-day-old plants) were harvested for total RNA isolation. (B) RNA blot analysis of mature miR171 and miR156 in stem (Int.2 from the bottom) of 35-day-old plants. (C) RNA blots of miR171 in 10-day-old *LOM* overexpression plants; *35S::Myc-rLOM1*/*2*/*3* promoted miR171 accumulation. (D) qRT-PCR analysis of the pri-miRNA transcripts of *MIR171A*, *B* and *C* in 10-day-old plants of indicated genotypes. All three pri-miRNA transcripts were up-regulated in *LUC-rLOM1OE* and reduced in *lomt* mutant plants. Error bars indicate s.d. (*n* = 3). Three biological replicates were analyzed, with similar results. (E) Expression of three *LOM* genes in 10-day-old plants of indicated genotypes. *LOM2* and *LOM3* were slightly reduced in *LUC-rLOM1OE* plants probably due to elevated miR171 accumulation. Error bars indicate s.d. (*n* = 3). Three biological replicates were analyzed, with similar results. (F) GUS staining of *MIR171A*/*B*/*C::GUS* in different backgrounds. The GUS activity was stronger in *LUC-rLOM1OE* and weaker in *MIR171BOE* plants than that in wild-type.

To see whether LOMs affected the SPL output through mediating miR156 accumulation, we compared the miR156 amount in wild-type, *lomt* and *35S::LUC-rLOM1* plants by RNA blots. Under long-day conditions, the miR156 accumulation was similar in 10-day-old seedlings (Figure 6A). Although slightly higher in 20-day-old *35S::LUC-rLOM1* plants, the miR156 level was not altered in the stem of *lomt* or *LUC-rLOM1OE* plants (Figure 6B), neither was *SPL9* expression (Figure S5). Thus it is unlikely that LOMs have a significant effect on miR156 abundance.

Interestingly, the miR171 level was clearly increased in *35S::LUC-rLOM1* but decreased in *lomt* mutant plants (Figure 6A), and overexpression of *LOM2* or *LOM3* also promoted miR171 accumulation (Figure 6C). qRT-PCR showed that the *MIR171A* transcript was much higher (up to 3-fold) in 35S::*LUC-rLOM1* but lower in *lomt* mutant than in wild-type plants, whereas the expression of *MIR171B* and *C* showed a less degree response to LOM1 (Figure 6D). *LOM2* and *LOM3* expression was slightly reduced in *35S::LUC-rLOM1* plants (Figure 6E), possibly due to the elevated miR171 levels. We also generated the *MIR171A*/*B*/*C::GUS* reporter lines and crossed them to *35S::LUC-rLOM1* and *35S::MIR171B* plants, respectively. Compared to wild-type, GUS activities of *MIR171A::GUS* and *MIR171B::GUS* were enhanced in 35S::*LUC-rLOM1* and weakened in *35S::MIR171B* plants, respectively (Figure 6F).

**Figure 7:**
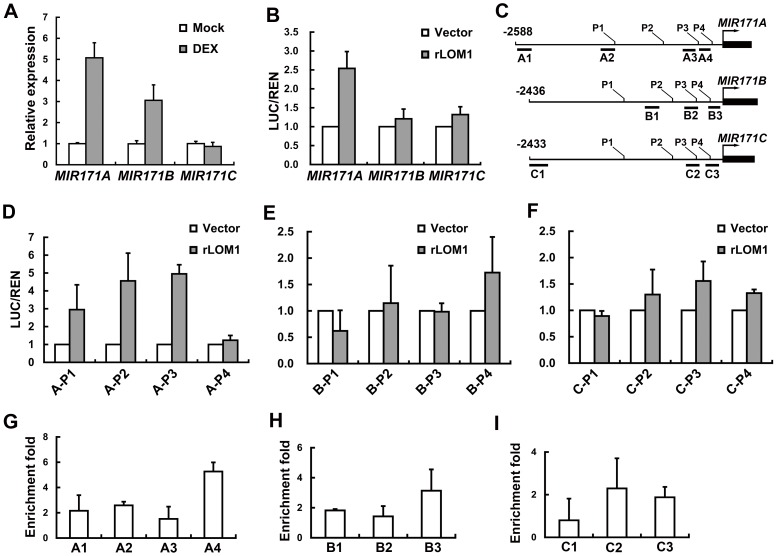
LOM1 directly binds *MIR171A* promoter. (A) Expression of *MIR171A*, *B* and *C* in rosette leaves of 10-day-old *35S::rLOM1-GR lomt* seedlings after a 4-h DEX or a mock treatment. Error bars indicate s.d. of three technical replicates, and the results were consistent in three biological replicates. (B) Relative reporter activities showing the induction of *MIR171* transcription by LOM1. *N. benthamiana* leaves were used for transient expression of the reporter (*MIR171A*/*B*/*C::LUC*) in combination with the effector (empty vector or *35S::rLOM1*). The relative LUC activities normalized to the REN activity are shown (LUC/REN, *n* = 3). (C) Schematic diagram of *MIR171A* (top), *MIR171B* (middle) and *MIR171C* (bottom) upstream genomic regions. Black box represents stem-loop of the pri-miR171; P1–P4 represent the start sites of truncated promoters used in dual-LUC assays in (D–F); A1–A4, B1–B3 and C1–C3 represent the DNA amplicons for ChIP assays in (G–I). (D–F) Relative activities of truncated promoters of *MIR171A* (D), *MIR171B* (E) and *MIR171C* (F) in response to LOM1 induction. (G–I) ChIP enrichment of *MIR171* promoter regions bound by Myc-LOM1. DNA fragments, isolated from rosette leaves of 10-day-old *35S::Myc-LOM1* and wild-type plants, were quantitatively analyzed by PCR, and *β-TUBULIN-2* promoter was used as a reference. Error bars indicate s.d. of three quantitative PCR replicates.

**Figure 8:**
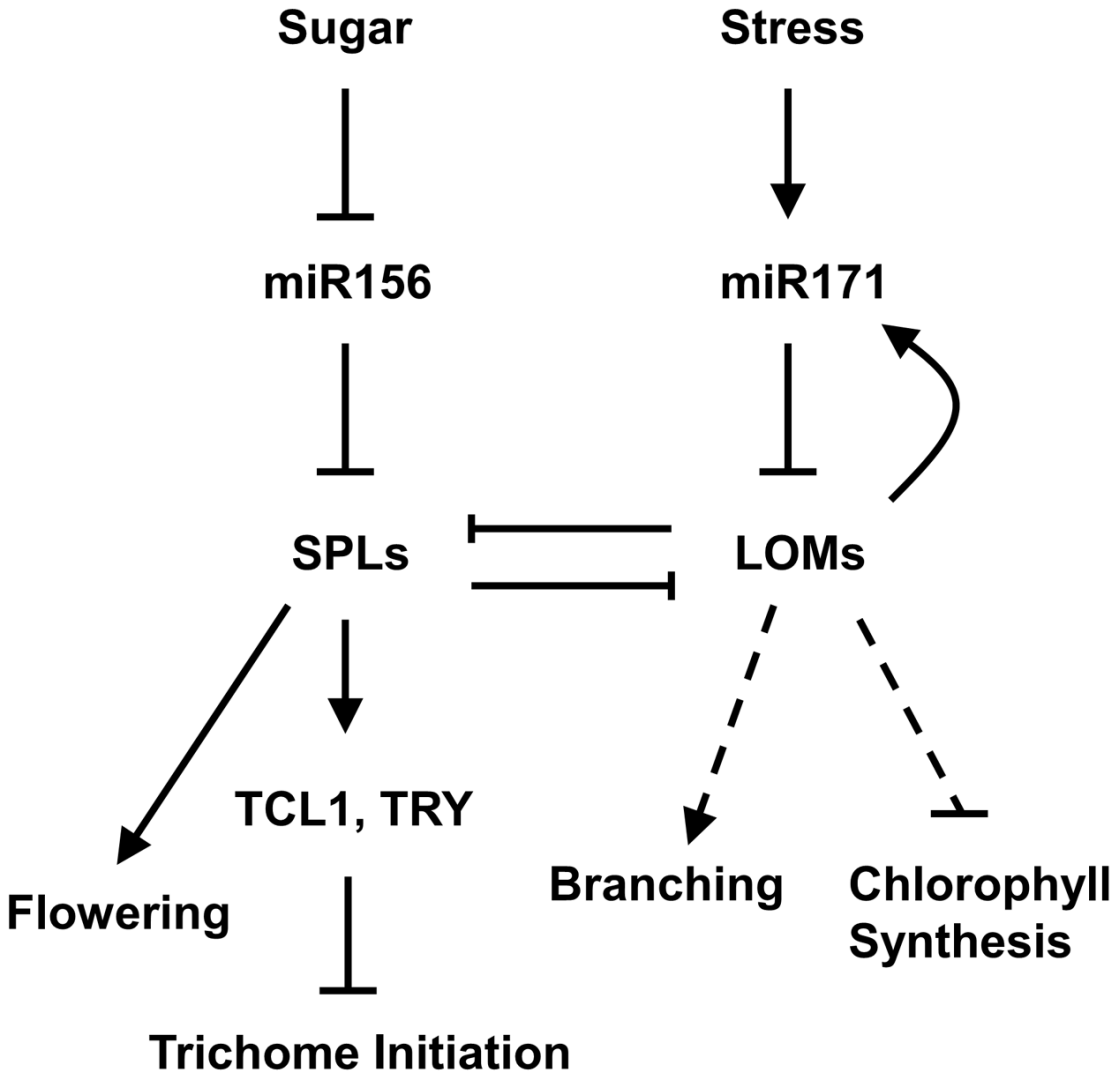
A model for miR171-LOM and miR156-SPL interaction in regulating trichome formation and other biological events. Trichomes are distributed on stem acropetally, with a high density at the basal part and sparse to nearly glabrous at apical part and inflorescence. The miR156-targeted SPLs up-regulate trichome negative factors *TRY* and *TCL1* to repress trichome production on stem and inflorescence, whereas miR171-targeted LOMs counteract the SPLs through protein-protein interaction. LOMs positively regulate *MIR171*, forming a feedback loop to maintain their homeostasis, which influences the SPL output. Arrows indicate positive regulation, blunt ends indicate negative regulation, and the dashed line indicates the unidentified pathway.

To investigate if *MIR171* genes were directly regulated by their targets, we used an inducible system to test the activity of LOM1 in activating *MIR171* expression. The 10-day-old *35S::rLOM1-GR lomt* seedlings were sprayed with 10 mM dexamethasone (DEX) to allow the translocation of LOM1-GR fusion protein into the nucleus. An obvious transcript increase of *MIR171A* and, to a less extent, of *MIR171B* was observed after 4 hours, whereas *MIR171C* was not induced during this period (Figure 7A). Furthermore, in transient assays using *N. benthamiana* leaves, the level of luciferase activity controlled by the *MIR171A* promoter was elevated significantly when LOM1 was co-expressed, while the *MIR171B* and *MIR171C* promoters exhibited a weak or marginal response (Figure 7B). To identify the LOM1 binding regions, we dissected the truncated promoters of the three *MIR171* genes (Figure 7C). Successive deletions from the 5′-end revealed a 143-bp promoter fragment of *MIR171A* (−201 to −343) which conferred the LOM1 induction (Figure 7D and Figure S6). However, the response of *MIR171B* and *MIR171C* promoters to LOM1 was negligible in *N. benthamiana* leaves (Figure 7E, 7F and Figure S6). Finally, we performed a chromatin immunoprecipitation (ChIP) assay using the 10-day-old *35S::Myc-rLOM1* plants, which showed LOM1 bound strongly to A4 region (Figure 7G), corresponding to the fragments identified in promoter deletion assays (Figure 7D and Figure S6). LOM1 weakly bound to promoters of *MIR171B* and *MIR171C* directly (Figure 7H and 7I). Together, these data reveal a regulatory feedback loop between LOMs and *MIR171* genes, particularly *MIR171A* (Figure 8), which explains the seemingly contradictory phenomenon that miR171 and its target *LOMs* show similar expression patterns and both are mounting to a high level in inflorescence (Figure S7).

## Discussion

By dissecting molecular mechanisms that control *Arabidopsis* trichome distribution, we found that the miR171-targeted LOMs directly interact with the miR156-targeted SPL9 and SPL2, leading to inhibition of the SPL activities. Remarkably, miR156 and miR171 function antagonistically in regulating many aspects of plant growth and development, including but far beyond the age-dependent trichome formation. Because both miR156 and miR171 are timing regulators, the interaction between their targets shed a new light on the endogenous network of plant aging. A similar but mechanically distinct scenario has been elucidated in *C. elegans*, where *lin-4* and *miR-239* oppositely regulate lifespan by common downstream genes [35], [36].

Except promoting the expression of miR172, SPLs positively regulate miR156 expression as well [3]. However, the miR171-LOM module is different from the miR156-SPL. The miR156 and its targets, such as SPL9, show reverse expression patterns [2]–[4], whereas miR171 and LOMs have a congruous temporal expression pattern (Figure S7). The level of LOMs elevates with age, leading to progressively activation of *MIR171* genes, which in turn keep the *LOM* transcripts under the fine control. This regulatory feedback loop ensures the homeostasis of miR171 and its targets. Furthermore, since the LOMs themselves are transcription factors, the effect of LOM-SPL interaction can be bilateral. Taken chlorophyll content as an example, miR156 overexpression suppresses chlorophyll biosynthesis and this suppression is LOM-dependent (Figure S8A and S8B). Several transgenic lines of *35S::rLOM1-YFP* and *35S::Myc-rLOM2* produced yellowish leaves during early vegetative stage due to decreased chlorophyll content as reported [26]. But this phenotype became less evident with plant aging (Figure S8C–S8E) when the SPL level was increasing, suggesting a possibility that the miR156-targeted SPLs enhance chlorophyll biosynthesis at least partially through negatively regulating the LOM activity. Finally, although overexpression of LOM1 and SPL9 simultaneously resulted in an intermediate phenotype, such as the trichome number shown in Figure 3, the two transcription factors may act independently on downstream genes. Identification of the targets of LOM factors will further our understanding of the biological significance of LOM-SPL interaction.

In addition to LOMs, DELLAs could bind to SPLs as well. DELLAs are degraded in response to gibberellin [37]–[39], resulting in release of the factors they bind, and the SPL-DELLA interaction integrates the hormone signals to the miR156-SPL pathway in regulating plant flowering [5]. MiR171 is not only regulated by endogenous cues, but also responds to environmental stress, such as cold, high salt and hydration [40]–[43]. Thus the LOM-SPL interaction may transduce environmental stimuli into endogenous signaling to adjust plant phase transition and development. Because both LOMs and SPLs mount with plant age, and their over-accumulation have adverse effects on plant growth and development [2], [3], [26], [44], we propose that plants have employed LOMs as a damper to measure the increasingly higher SPL output at protein level in aged plants when the miR156 level is low, and likewise SPLs may temporally restrict the activity of LOMs. Since the miR156-targeted SPLs function as a key endogenous age cue and SPL9 is one of the highly active SPL members, the LOM-SPL interaction has a profound contribution to programming plant life.

Mining of genome data revealed that both miR156 and miR171 are highly conserved in land plants from moss (*Physcomitrella patens*) to flowering plants of both monocots and dicots [45], and in crop plants they control important agronomic traits [46], [47]. In the diploid cotton species of *Gossypium raimondii* there are 15 *MIR171* and seven putative *LOM* genes, of which five *LOMs* contain the miR171 recognition sites (see [48] and Figure S9). It would be interesting to examine if LOMs are involved in regulating cotton fiber (seed trichome) development. Notably miR171a* is also functional in gene silencing and the miR171a*-*SU(VAR)3-9 HOMOLOG8* pair was proposed to have evolved very recently in the *Arabidopsis* lineage [44]. A recent report showed that overexpression of miR171 (*Hvu-pri-miR171a*) in barley up-regulated miR156 and repressed vegetative phase transitions [49], which is in contrast with the opposite effects of the two miRNAs in *Arabidopsis* described herein and reported by others [26], [29], [50]. Whether the interplay between the two conserved aging miRNAs varies with plant taxa is a subject of further study.

## Materials and Methods

### Plant materials and constructs

Plants of *Arabidopsis thaliana*, ecotype Columbia (Col-0), and *Nicotiana benthamiana* were grown at 22°C in long days (16 h light/8 h dark). The *lom* triple mutant [26], *35S::LUC-rLOM1* [26], *35S::MIR156F* [23], *35S::MIM156* [23], *SPL9::GFP-rSPL9* [23], *TCL1::GUS* [23], *TCL1mu3*/*4::GUS* [23] have been described previously. The *gl1* (SALK_039478), *try* (SALK_029760), *tcl1* (SALK_055460) and *gl3 egl3* (CS66490) mutants were obtained from Arabidopsis Biological Resource Center (ABRC).

For *MIR171A*/*B*/*C::GUS* constructs, the promoters of *MIR171A*/*B*/*C* (∼2 kb) were PCR amplified using PrimeSTAR HS DNA polymerase (TaKaRa) and individually fused to the GUS coding region. For LOM constructs, the miRNA-resistant versions were created by two-round PCR. The resultant fragment was inserted into a vector which harbors a *35S::6*×*Myc* cassette to generate *35S::Myc-rLOM1*/*2*/*3*. Then the *35S* promoter was replaced by a native promoter to generate *LOM1::Myc-rLOM1* and *LOM12::Myc-rLOM2*. At least 30 T1 seedlings were analyzed for each construct. For BiFC constructs, coding regions of the miRNA-resistant *LOMs* and *SPL9* were PCR amplified and cloned into JW771 and JW772 [51], respectively. Primers are listed in Supplementary Table S1.

### Trichome number and flowering time measurement

The trichome numbers were counted on each internode of stem, and the number was divided by the length of each internode to calculate the trichome density (number per centimeter). Flowering time was measured by counting the total number of leaves (rosette and cauline leaves) and the number of days to flowering under long-day condition. The data were given as mean s.d. and analyzed by *t* test.

### Gene expression analyses

RNAs were extracted with Trizol reagent (Invitrogen) following the manufacturer's instructions. Total RNAs of 1 µg were used for reverse transcription in a 20 µL reaction system with M-MLV Reverse Transcriptase kit (Invitrogen). The fragments of interest were amplified by RT-PCR using sequence-specific primers (see Supplementary Table S1). Real-time PCR was performed with SYBR Premix Ex Taq II (Takara), and amplification was monitored on the Mastercycler ep RealPlex2 (Eppendorf). The gene expression level was normalized to reference gene *β-TUBULIN2* (At5g62690). For DEX induction, 10-day-old *35S::rLOM1-GR lomt* seedlings were sprayed with 10 mM DEX (Sigma-Aldrich) or alcohol (mock control). After 4 hours, rosette leaves were harvested.

### Protein–protein interaction assays

For subcellular localization assay, *35S::GFP-rSPL9* and *35S::mCherry-rLOM1* were transiently expressed in *N. benthamiana* leaves. After 3 days, the materials were observed using confocal microscope OLYMPUS FV1000.

Yeast two-hybrid assay was performed using the Matchmaker GAL4 Two-Hybrid System according to the manufacturer's manual (Clontech). Full-length or truncated cDNAs of LOM1 were inserted into pGBKT7 and those of SPLs into pGADT7, respectively. Plasmids were transferred into yeast strain AH109 (Clontech) by the LiCl-PEG method. The interactions were tested on SD/-Leu/-Trp/-His plates supplemented with 15 mM 3-amino-1,2,4,-triazole. Three independent clones for each transformation were tested.

BiFC assays were performed as described [51], [52]. Briefly, four chimerical constructs were used. SPL9 was fused to C-terminal half of luciferase (cLUC-rSPL9) and three LOMs to the N-terminal half (LOMs-nLUC); cLUC and nLUC alone were used as controls. *Agrobacterium tumefaciens* cells were re-suspended in infiltration buffer (10 mM MgCl_2_, 10 mM MES pH 5.7, 150 µm acetosyringone) at OD_600_ = 0.8. *35S::P19-HA* and the suspension was co-infiltrated to inhibit gene silencing [53]. After 3 days, a total of 0.8 mM luciferin was infiltrated and the LUC activity was monitored. The following pairs of constructs were used for co-infiltration: cLUC-rSPL9 and LOMs-nLUC, cLUC and LOMs-nLUC, cLUC-rSPL9 and nLUC, as well as cLUC and nLUC.

### Small RNA blot

Total RNAs were extracted using Trizol reagent (Invitrogen), and 5–20 µg of the total RNA were resolved on 17% polyacrylamide gels under denaturing conditions (7 M urea). RNAs were then transferred to HyBond-N+ membranes (GE Healthcare) by semidry blotting, and membranes were hybridized with oligonucleotide DNA probes labeled with digoxigenin using the DIG Oligonucleotide 3-End Labeling Kit, Second Generation (Roche). Oligonucleotide sequences are listed in Supplementary Table S1 online.

### ChIP assays

Chromatin immunoprecipitation (ChIP) experiments were performed as described [54]. Tissues (∼2 g) of 10-day-old *35S::Myc-rLOM1* or wild-type seedlings were harvested and then cross-linked in formaldehyde solution (1%) under a vacuum. The material was washed and ground in liquid nitrogen, the resultant powder was re-suspended in extraction buffer (0.4 M sucrose, 10 mM Tris-HCl, pH 8.0, 10 mM MgCl_2_, 5 mM mercaptoethanol, 0.1 mM PMSF, and 1× protease inhibitor [Roche]) and lysis buffer (50 mM HEPES, pH 7.5, 150 mM NaCl, 1 mM EDTA, 1% Triton X-100, 0.1% deoxycholate sodium, and 0.1% SDS), successively, followed by sonification (output 3, 6×10 s). An anti-Myc antibody (Abmart) was added for precipitation. After several washes, DNA samples were reversely cross-linked and then purified using a PCR purification kit (Qiagen). The relative amounts of the DNA amplicons were analyzed by quantitative PCR using the *β-TUBULIN2* gene promoter as a reference. Relative enrichment was calculated by normalizing the value in *35S::Myc-rLOM1* against the value in wild-type.

### Dual-LUC assays

A dual-luc method using *N. benthamiana* plants was used [55]. Briefly, the effector plasmid is *35S::rLOM1* or empty vector, and the reporter plasmid, pGreen-0800-LUC, harbors two luciferases: the firefly luciferase (LUC) controlled by the *MIR171* promoter, and the *Renilla* (REN) luciferase controlled by the constitutive *35S* promoter. The *Agrobacterium* strain containing the reporter was mixed with the effector strain (at the reporter:effector ratio of 1∶3). The mixture was infiltrated into leaves of *N. benthamiana*. Three days later, leaf samples were collected for the dual-luc assay using commercial Dual-Luciferase Reporter Assay System (Promega) according to the manufacturer's instruction. The LUC activity was normalized to REN. Three biological repeats were measured for each sample.

### GUS staining

GUS activity was assayed by staining. Plant materials were submerged in 0.5 mg/mL X-Gluc solution (0.1 M sodium phosphate buffer, pH 7.0, 10 mM EDTA, 0.1% Triton X-100, 0.5 mM potassium ferrocyanide, 0.5 mM potassium ferricyanide), vacuumized and kept at 37°C. Subsequent materials were decolorized in 70% ethanol.

### Accession numbers

*LOM1* (At2G45160), *LOM2* (At3G60630), *LOM3* (At4G00150), *MIR171A* (At3G51375), *MIR171B* (At1G11735), *MIR171C* (At1G62035), *SPL9* (At2g42200), *SPL2* (At5G43270), *MIR156F* (At5G26147), *SOC1* (At2g45660), *GL1* (At3G27920), *GL3* (At5G41315), *EGL3* (At1G63650), *TCL1* (At2g30432), *TRY* (At5G53200), *GIS* (At3g58070), *GIS2* (At5g06650 ), *ZFP8* (At2g41940), *β-TUBULIN-2* (At5g62690) and *Ph-HAM* (AY112704).

## Supporting Information

Figure S1 **Expression of trichome regulator genes.** (A) qRT-PCR analysis of expression of trichome promoting genes in wild-type, *lomt* mutant and *LUC-rLOM1OE* plants. Expression of *GIS*, *GIS2*, *ZFP8* and *GL3* did not change evidently. (B and C) Expression of trichome repressor genes in first internode (B) and inflorescence (C). Expression of *CPC*, *ETC1* and *TCL2* did not change in *lomt* and *LUC-rLOM1OE* plants in first internode; *CPC* was higher in *LUC-rLOM1OE* inflorescence.
(TIF)

Figure S2 ***LOM1OE*** **and** ***MIR156FOE*** **plants show similar phenotypes.** View of 49-day-old plants of the indicated genotypes. Plants were grown under short-day condition (8 h light/16 h dark). Note that *LOM1OE* (*35S::LUC-rLOM1*) and *MIR156FOE* (*35S::MIR156F*) plants were both yellow-green; on the contrary, *lomt* mutant and *SPL* accumulation plants (*35S::MIM156* and *SPL9::GFP-rSPL9*) were dark-green. *35S::LUC-rLOM1* and *35S::MIR156F* plants produced more rosette leaves than wild-type. Bar = 2 cm.
(TIF)

Figure S3 **LOMs accumulation delays flowering.** (A) SPL2 interacts with three LOMs in yeast. (B–D) Flowering time of wild-type (B), *lomt* (C) and *35S::LUC-rLOM1* (D) plants under long-day conditions. The *x* axis indicates the number of days after germination and the *y* axis indicates percentage of plants that flowered on a given day. (E) Flowering time of the plants indicated in (B–D). The number of days to flowering was counted when the first floral bud opens. Data are given as mean s.d. and analyzed by *t* test. **P<0.01, relative to the wild-type; *n*, number of plants analyzed.
(TIF)

Figure S4 **Alignment of N-terminus of three LOMs of** ***Arabidopsis thaliana*** **.** LOM1N and LOM2N are more similar in length and amino acid sequence identity.
(TIF)

Figure S5 **Expression of** ***SPL9*** **in seedling and inflorescence.** Analysis by qRT-PCR shows that expression of *SPL9* did not change evidently in *lomt* and *35S::LUC-rLOM1* plants in either seedling or inflorescence.
(TIF)

Figure S6 **Identification of promoter regions of** ***MIR171*** **genes responsible for LOM1 induction.** (A) LUC activities driven by different upstream fragments of the *MIR171A* gene. Nucleotides are numbered from the stem-loop of the pri-miRNA. The *Agrobacterium* strain containing the *MIR171::LUC* reporter combined with the effector strain containing the *35S::rLOM1* or the empty vector were infiltrated into *N. benthamiana* leaves. After 3 days, luciferin was infiltrated into the same region and the LUC activities were monitored. Note that LUC activities were increased when *MIR171A::LUC* was combined with *35S::rLOM1*, in comparison with the empty vector control. Because A-P3 fragment still responded to LOM1 while A-P4 did not, the LOM1-binding motifs are located in the region between −343 and −201. (B and C) LUC activities driven by truncated promoters of *MIR171B* (B) and *MIR171C* (C) in response to LOM1.
(TIF)

Figure S7 ***SPL9*** **,** ***LOMs*** **and** ***MIR171*** **show similar temporal expression pattern.** (A) GUS staining of *LOM1::rLOM1-GUS* plants. GUS activity in newly emerged leaves was stronger than in elder ones. (B) Relative expression level of *SPL9* and three *LOMs* in stem and inflorescence. Based on qRT-PCR, expression of *SPL9* and three *LOMs* increased gradually along the stem from bottom to top (Int.1 to Int.3) and reached the highest level in inflorescence (Inf.). The expression level of each gene in Int.1 was set to 1. (C) *MIR171* expression in main stem and inflorescence. Transcript abundance of *pri-MIR171A*, *B* and *C* increased gradually along the main stem from basal to apex and reached the highest levels in inflorescence. The expression level of each gene in Int.1 was set to 1. (D) The mature miR171 level was increasing with age, and the miR156 showed an opposite accumulation pattern. *U6* was used as an internal reference.
(TIF)

Figure S8 **LOM-SPL module plays a role in chlorophyll synthesis.** (A) View of wild-type, *35S::MIR156F*, *35S::MIR171B* and *35S::MIR156F*×*35S::MIR171B* plants. Plants were grown in long-day condition for 20 days. Bar = 1 cm. (B) Chlorophyll content of the genotypes shown in (A). FW, fresh weight. **P<0.01 compared with wild-type. Chlorophyll was measured as described previously [56]. (C–E) View *35S::Myc-rLOM2* transgenic *Arabidopsis* at indicated age under long day condition. This transgenic line showed severe development defects, including up-curly yellowish leaves, soft stem, late flowering and infertility. Arrows in (D) indicate one leaf exhibiting different colors. After the transition shown in (D), the newly emerged leaves showed a wild-type shape and color, suggesting normal chlorophyll content (E). Stars in (E) indicate the elder up-curly yellow-green rosette leaves. Scale bars, 1 cm.
(TIF)

Figure S9 **MiR171s and LOMs in** ***Gossypium raimondii*** **.** (A) Secondary structure of cotton miR171 predicted precursors. Sequences (80–200 bp) of 15 potential pri-miR171s derived from *G. raimondii* genome were analyzed using RNAfold program. (B) Phylogeny of LOM proteins. This unrooted phylogenetic tree of 12 LOM proteins was generated using Neighbour-joining method without distance corrections in Clustalw2 web service of EMBL-EBI. Sequences include three LOMs, At-HAM4 (At4G36710) from *Arabidopsis thaliana*, Ph-HAM (AY112704) from *Petunia hybrida* and seven cotton LOMs from *Gossypium raimondii*.
(TIF)

Table S1 **Oligonucleotide primers used in the study.**
(DOCX)
