# Supplementary figures and images for: Interaction between Two Timing MicroRNAs Controls Trichome Distribution in Arabidopsis
Source: PLoS Genet. 2014 Apr 3;10(4):e1004266. doi: 10.1371/journal.pgen.1004266 (PMC3974651; doi:10.1371/journal.pgen.1004266)

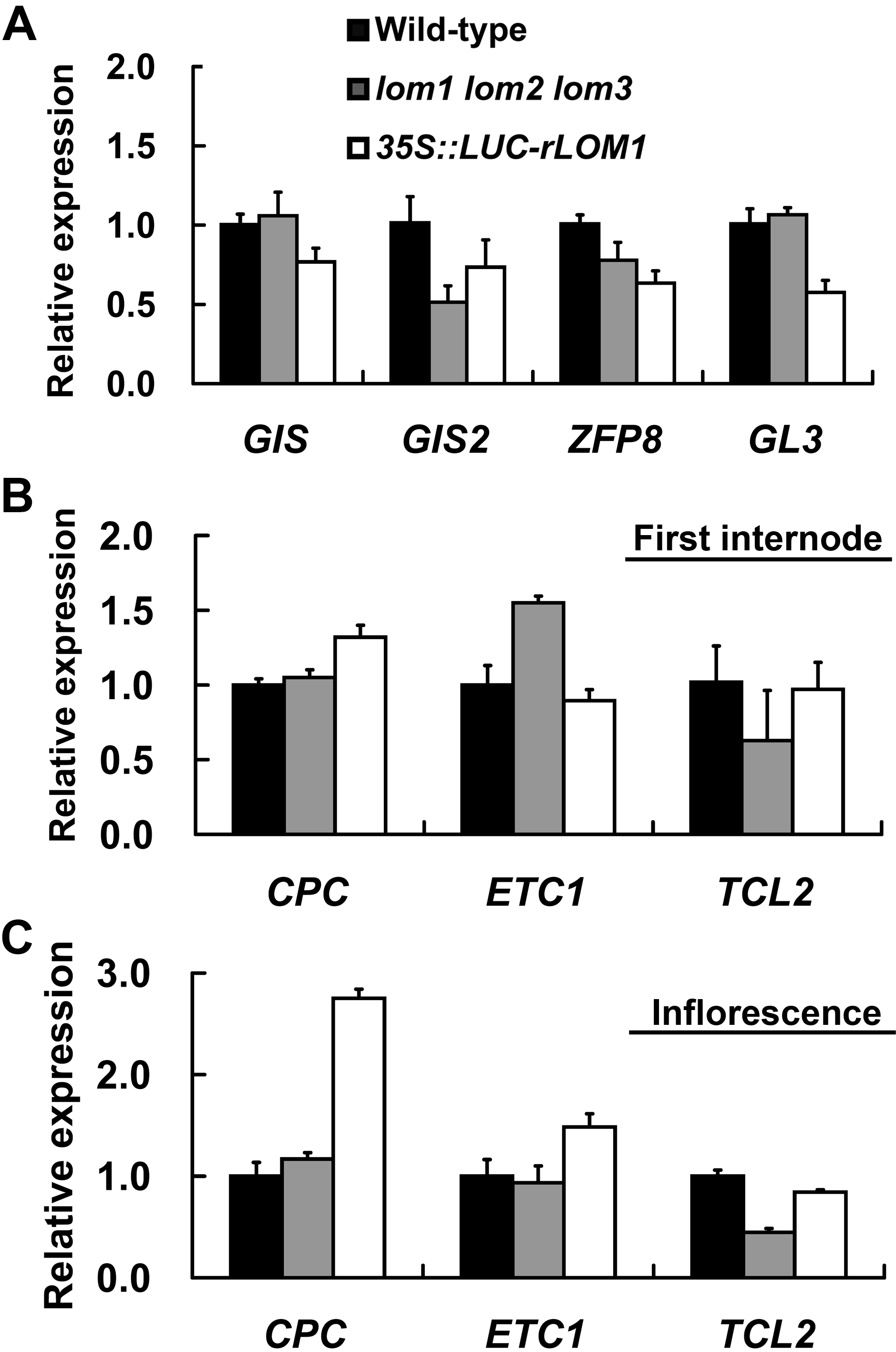

Supplement: Figure S1 — Expression of trichome regulator genes. (A) qRT-PCR analysis of expression of trichome promoting genes in wild-type, lomt mutant and LUC-rLOM1OE plants. Expression of GIS, GIS2, ZFP8 and GL3 did not change evidently. (B and C) Expression of trichome repressor genes in first internode (B) and inflorescence (C). Expression of CPC, ETC1 and TCL2 did not change in lomt and LUC-rLOM1OE plants in first internode; CPC was higher in LUC-rLOM1OE inflorescence. (TIF) [file pgen.1004266.s001.tif]

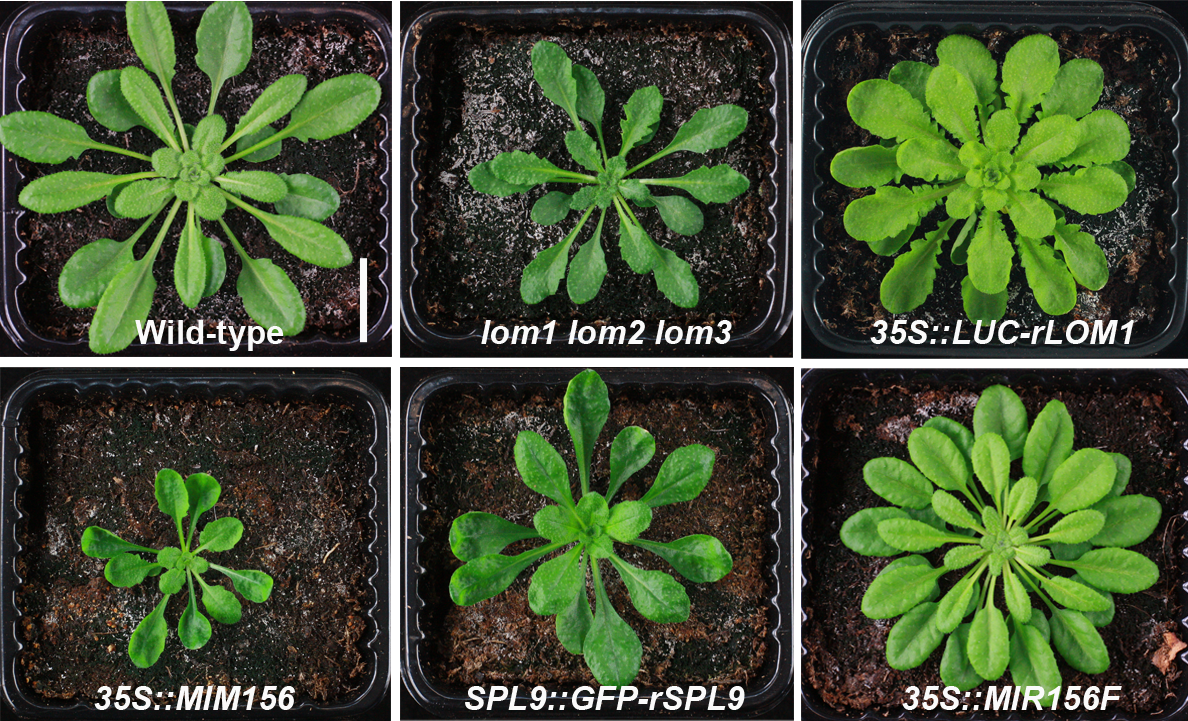

Supplement: Figure S2 — LOM1OE and MIR156FOE plants show similar phenotypes. View of 49-day-old plants of the indicated genotypes. Plants were grown under short-day condition (8 h light/16 h dark). Note that LOM1OE (35S::LUC-rLOM1) and MIR156FOE (35S::MIR156F) plants were both yellow-green; on the contrary, lomt mutant and SPL accumulation plants (35S::MIM156 and SPL9::GFP-rSPL9) were dark-green. 35S::LUC-rLOM1 and 35S::MIR156F plants produced more rosette leaves than wild-type. Bar = 2 cm. (TIF) [file pgen.1004266.s002.tif]

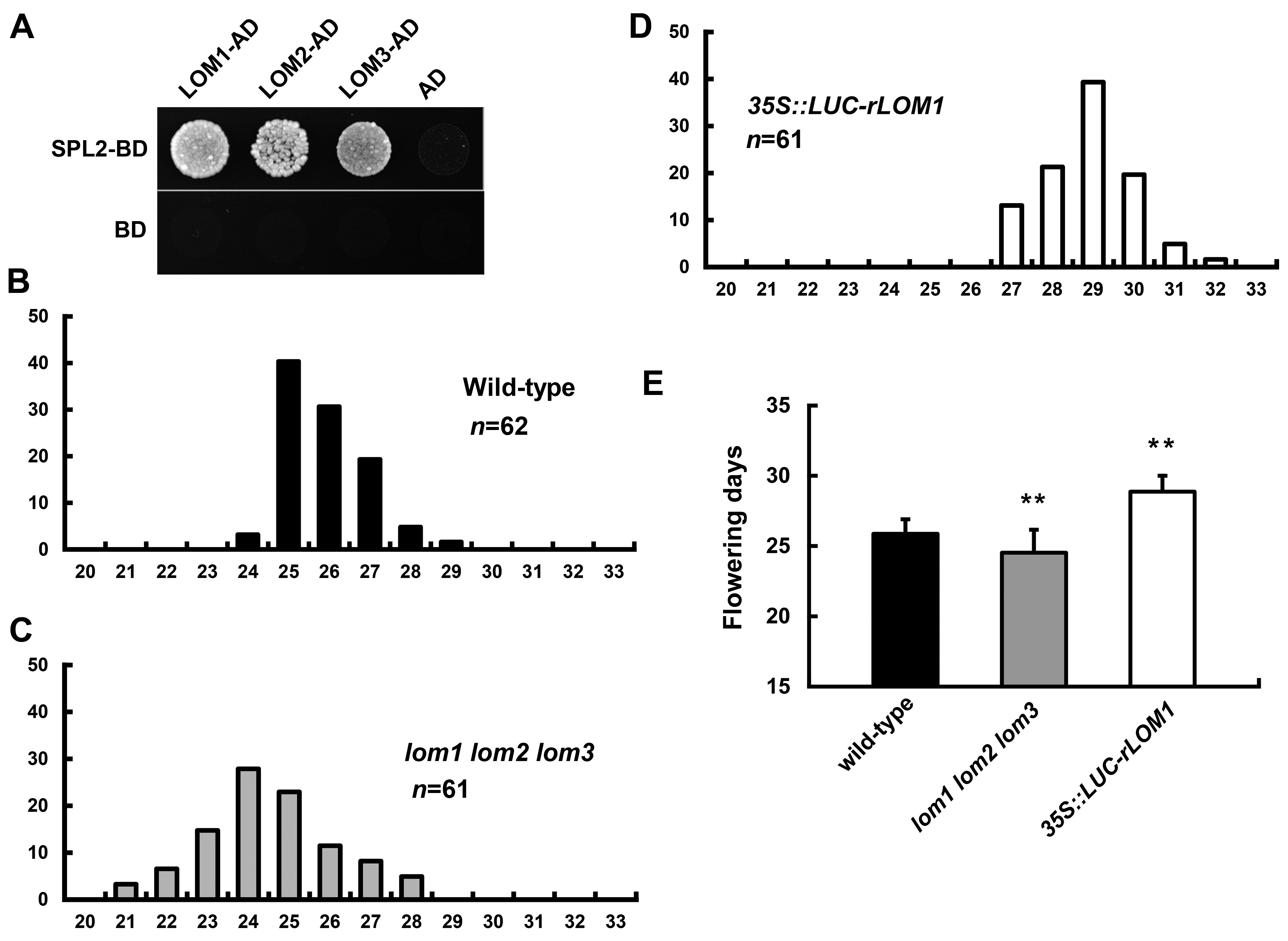

Supplement: Figure S3 — LOMs accumulation delays flowering. (A) SPL2 interacts with three LOMs in yeast. (B–D) Flowering time of wild-type (B), lomt (C) and 35S::LUC-rLOM1 (D) plants under long-day conditions. The x axis indicates the number of days after germination and the y axis indicates percentage of plants that flowered on a given day. (E) Flowering time of the plants indicated in (B–D). The number of days to flowering was counted when the first floral bud opens. Data are given as mean s.d. and analyzed by t test. **P<0.01, relative to the wild-type; n, number of plants analyzed. (TIF) [file pgen.1004266.s003.tif]

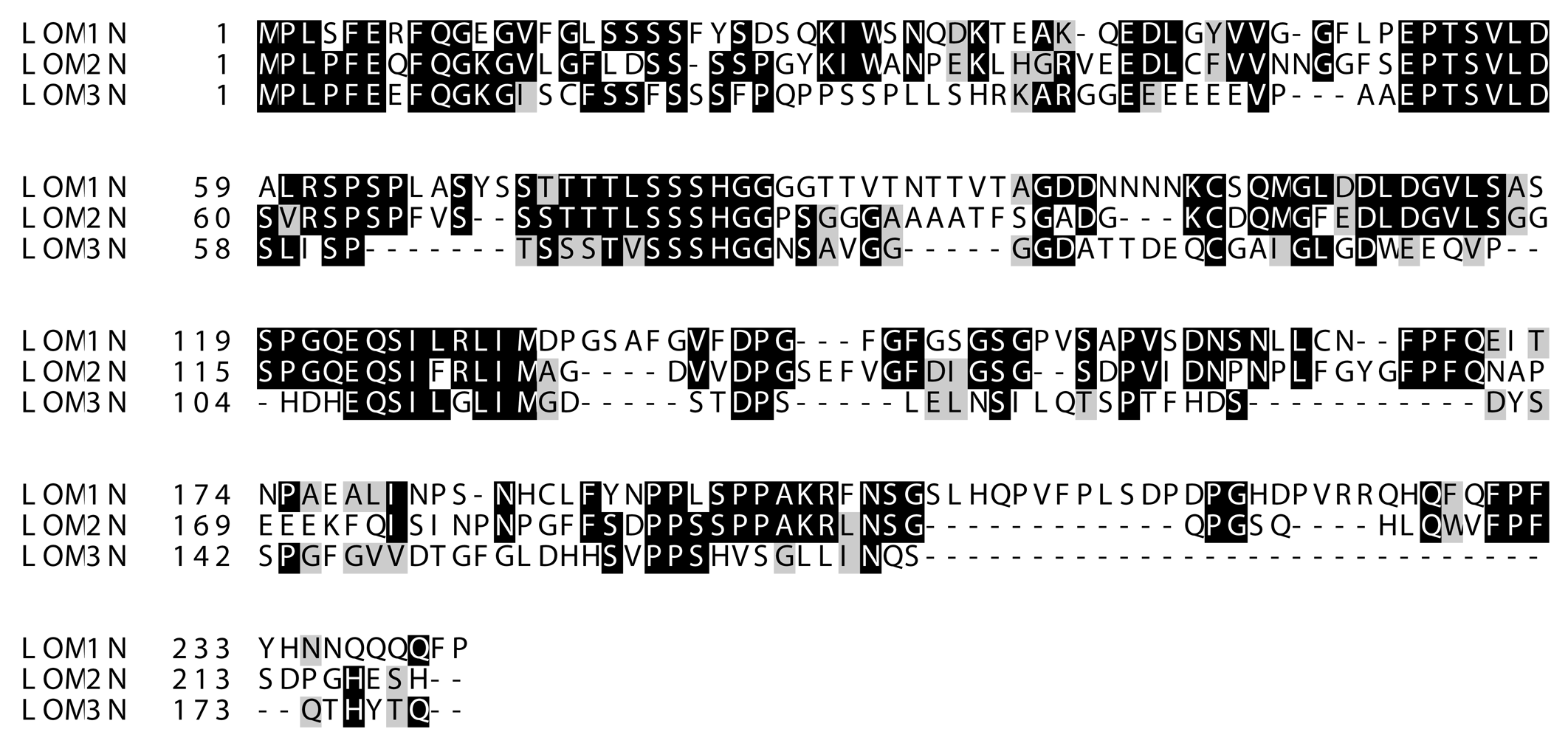

Supplement: Figure S4 — Alignment of N-terminus of three LOMs of Arabidopsis thaliana . LOM1N and LOM2N are more similar in length and amino acid sequence identity. (TIF) [file pgen.1004266.s004.tif]

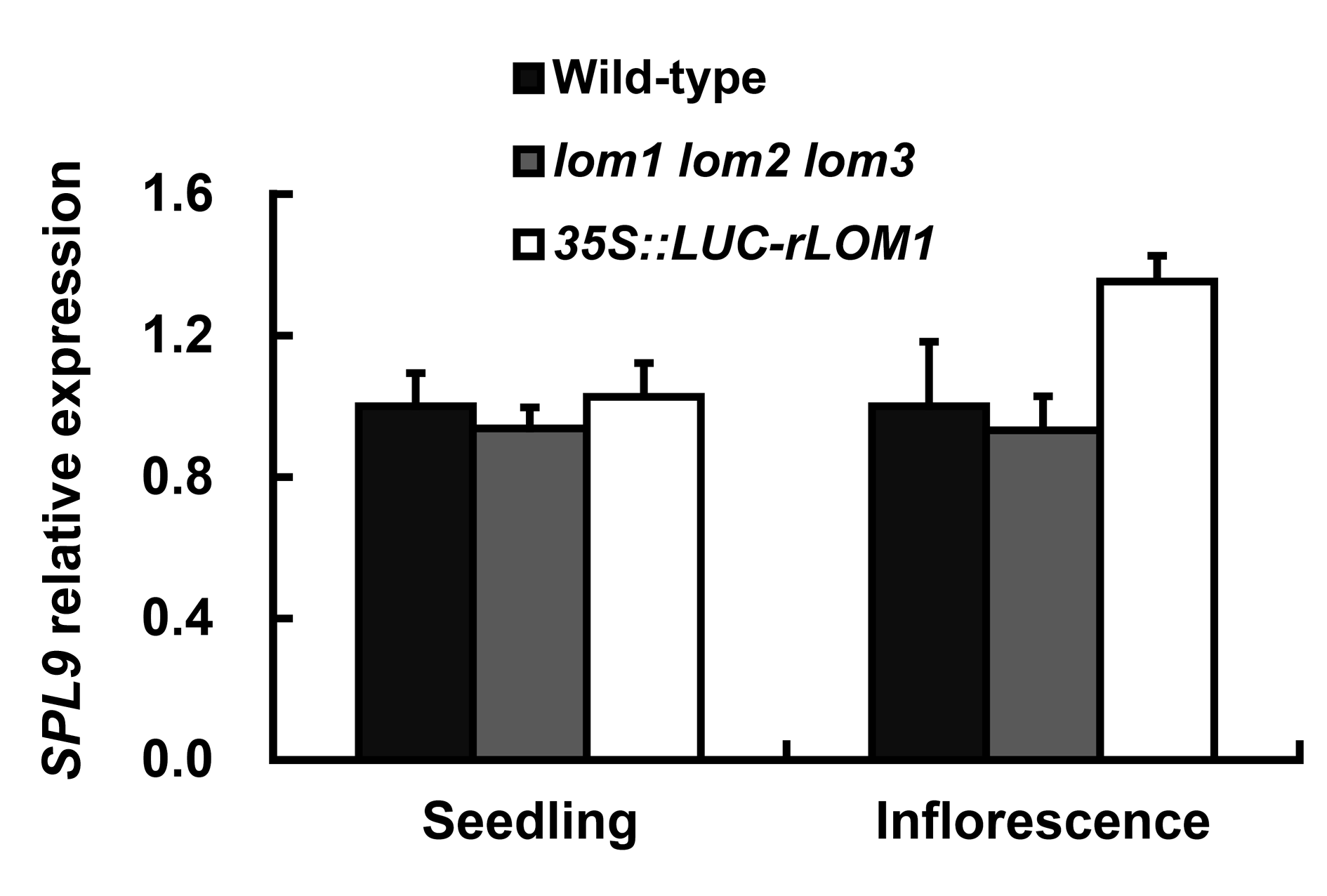

Supplement: Figure S5 — Expression of SPL9 in seedling and inflorescence. Analysis by qRT-PCR shows that expression of SPL9 did not change evidently in lomt and 35S::LUC-rLOM1 plants in either seedling or inflorescence. (TIF) [file pgen.1004266.s005.tif]

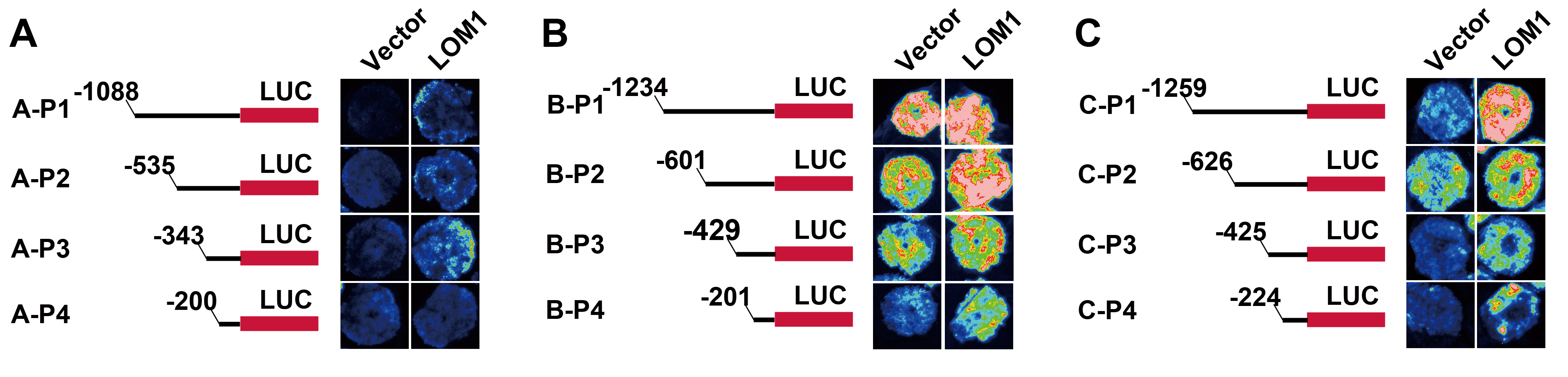

Supplement: Figure S6 — Identification of promoter regions of MIR171 genes responsible for LOM1 induction. (A) LUC activities driven by different upstream fragments of the MIR171A gene. Nucleotides are numbered from the stem-loop of the pri-miRNA. The Agrobacterium strain containing the MIR171::LUC reporter combined with the effector strain containing the 35S::rLOM1 or the empty vector were infiltrated into N. benthamiana leaves. After 3 days, luciferin was infiltrated into the same region and the LUC activities were monitored. Note that LUC activities were increased when MIR171A::LUC was combined with 35S::rLOM1, in comparison with the empty vector control. Because A-P3 fragment still responded to LOM1 while A-P4 did not, the LOM1-binding motifs are located in the region between −343 and −201. (B and C) LUC activities driven by truncated promoters of MIR171B (B) and MIR171C (C) in response to LOM1. (TIF) [file pgen.1004266.s006.tif]

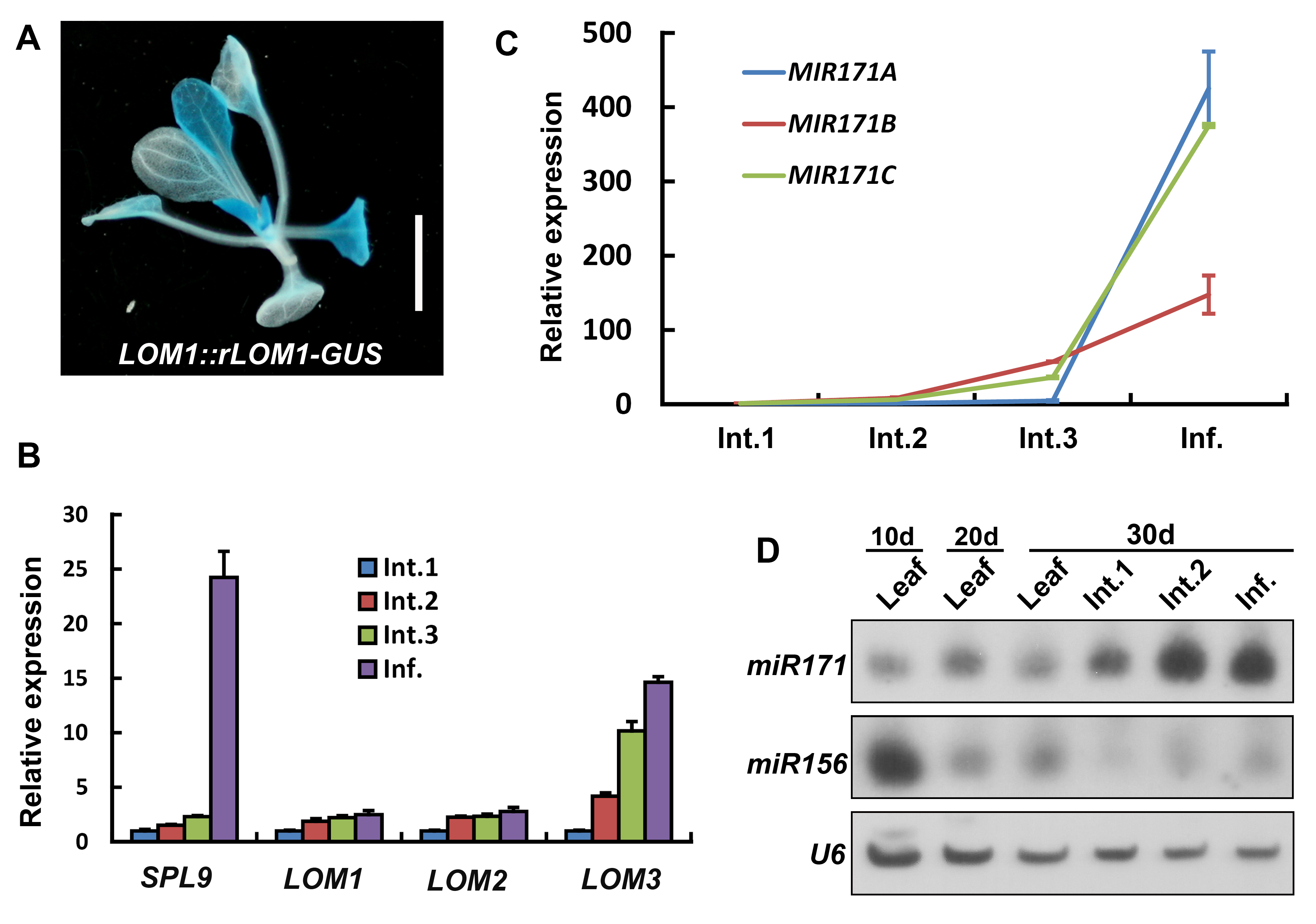

Supplement: Figure S7 — SPL9 , LOMs and MIR171 show similar temporal expression pattern. (A) GUS staining of LOM1::rLOM1-GUS plants. GUS activity in newly emerged leaves was stronger than in elder ones. (B) Relative expression level of SPL9 and three LOMs in stem and inflorescence. Based on qRT-PCR, expression of SPL9 and three LOMs increased gradually along the stem from bottom to top (Int.1 to Int.3) and reached the highest level in inflorescence (Inf.). The expression level of each gene in Int.1 was set to 1. (C) MIR171 expression in main stem and inflorescence. Transcript abundance of pri-MIR171A, B and C increased gradually along the main stem from basal to apex and reached the highest levels in inflorescence. The expression level of each gene in Int.1 was set to 1. (D) The mature miR171 level was increasing with age, and the miR156 showed an opposite accumulation pattern. U6 was used as an internal reference. (TIF) [file pgen.1004266.s007.tif]

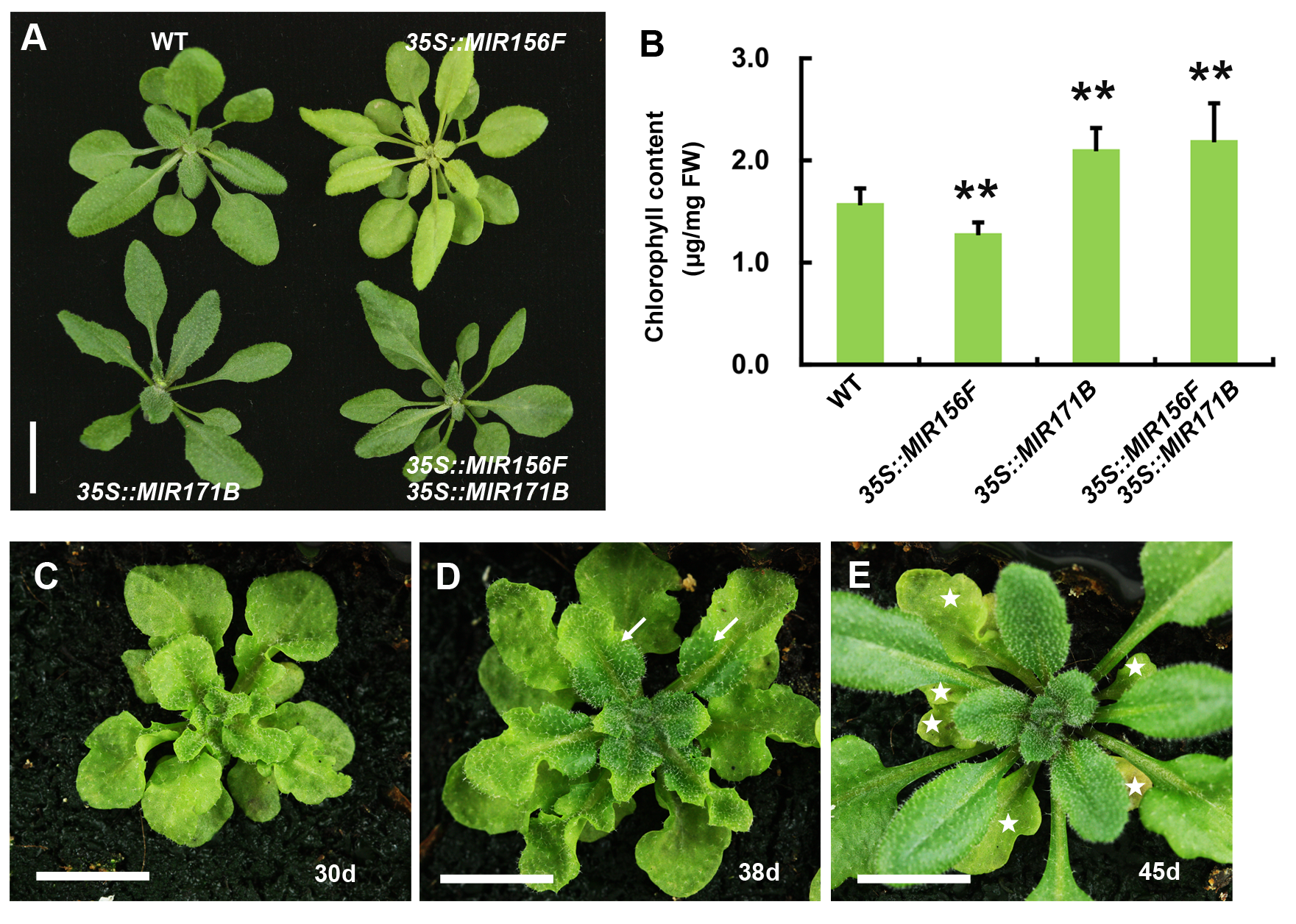

Supplement: Figure S8 — LOM-SPL module plays a role in chlorophyll synthesis. (A) View of wild-type, 35S::MIR156F, 35S::MIR171B and 35S::MIR156F×35S::MIR171B plants. Plants were grown in long-day condition for 20 days. Bar = 1 cm. (B) Chlorophyll content of the genotypes shown in (A). FW, fresh weight. **P<0.01 compared with wild-type. Chlorophyll was measured as described previously [56]. (C–E) View 35S::Myc-rLOM2 transgenic Arabidopsis at indicated age under long day condition. This transgenic line showed severe development defects, including up-curly yellowish leaves, soft stem, late flowering and infertility. Arrows in (D) indicate one leaf exhibiting different colors. After the transition shown in (D), the newly emerged leaves showed a wild-type shape and color, suggesting normal chlorophyll content (E). Stars in (E) indicate the elder up-curly yellow-green rosette leaves. Scale bars, 1 cm. (TIF) [file pgen.1004266.s008.tif]

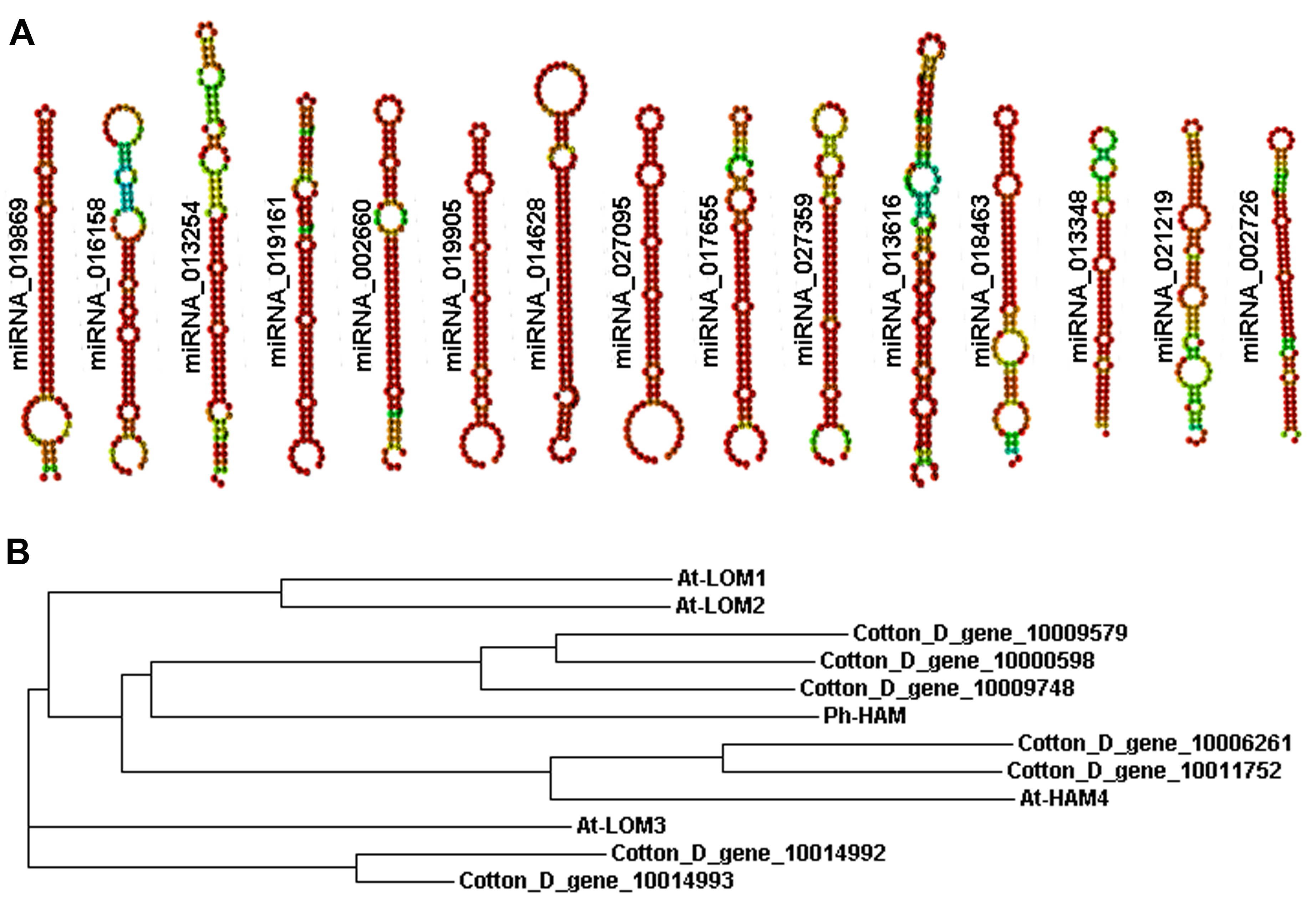

Supplement: Figure S9 — MiR171s and LOMs in Gossypium raimondii . (A) Secondary structure of cotton miR171 predicted precursors. Sequences (80–200 bp) of 15 potential pri-miR171s derived from G. raimondii genome were analyzed using RNAfold program. (B) Phylogeny of LOM proteins. This unrooted phylogenetic tree of 12 LOM proteins was generated using Neighbour-joining method without distance corrections in Clustalw2 web service of EMBL-EBI. Sequences include three LOMs, At-HAM4 (At4G36710) from Arabidopsis thaliana, Ph-HAM (AY112704) from Petunia hybrida and seven cotton LOMs from Gossypium raimondii. (TIF) [file pgen.1004266.s009.tif]
