# Supplementary material for: Interaction between Two Timing MicroRNAs Controls Trichome Distribution in Arabidopsis
Source: PLoS Genet. 2014 Apr 3;10(4):e1004266. doi: 10.1371/journal.pgen.1004266 (PMC3974651; doi:10.1371/journal.pgen.1004266)
Supplement: Table S1 — Oligonucleotide primers used in the study. (DOCX) [file pgen.1004266.s010.docx]

Supplementary Table S1 Oligonucleotide Primers

| **Primer** | **Sequence (5’-3’)** | **Purpose** |
| --- | --- | --- |
| Tubulin-F | TCAAGAGGTTCTCAGCAGTA | qRT-PCR |
| Tubulin-R | TCACCTTCTTCATCCGCAGTT | qRT-PCR |
| TCL1-F | AAACCGTCTTCGCCGCCTTCA | qRT-PCR |
| TCL1-R | TCCTTTGCCTCACGTCCCACCA | qRT-PCR |
| TRY-F | TTGTCGGTGATAGGTGGGATTT | qRT-PCR |
| TRY-R | ACGGTGAGGCTTGGTATGTTTG | qRT-PCR |
| SPL9-F | GGCGACTCAAACTGTGCTCT | qRT-PCR |
| SPL9-R | CATTGCCGTGCCACTACTTAT | qRT-PCR |
| MIR171A-F | GATATTGGCCTGGTTCACTC | qRT-PCR |
| MIR171A-R | CCACAAAGTCCAAAATAGAG | qRT-PCR |
| MIR171B-F | GGAGCTAAGTGGAGATTATAG | qRT-PCR |
| MIR171B-R | GGTTATAATAACTATCTTTGCC | qRT-PCR |
| MIR171C-F | GATGGAGTTTGGTGTAATAAG | qRT-PCR |
| MIR171C-R | CACATCAACTAACATAGAATC | qRT-PCR |
| LOM1-F1 | ACAACAATTCCCGTCGTCTT | qRT-PCR |
| LOM1-R1 | GAGACGACCATTGTCCTCCA | qRT-PCR |
| LOM2-F2 | TCCGGTTACCTCCCATTGATAC | qRT-PCR |
| LOM2-R2 | CTTCCGCATTATTCAGATTCCC | qRT-PCR |
| LOM3-F2 | GACCTCCTCGGCTCCATCTCGT | qRT-PCR |
| LOM3-R2 | GTCCGTTGGACTAAACACTCG | qRT-PCR |
| SOC1-F | ATAGGAACATGCTCAATCGAGGAGCTG | qRT-PCR |
| SOC1-R | TTTCTTGAAGAACAAGGTAACCCAATG | qRT-PCR |
| 171Apro-F-PstI | AACTGCAGACATAATGTGAGTATAGAGTCTG | promoter GUS fusion |
| 171Apro-R-SmaI | TCCCCCGGGCAAAGGGACTCTCTCATGCTTAAAG | promoter GUS fusion |
| 171Bpro-F-PstI | AACTGCAGGTTGGAAACGAACTAACTTGAG | promoter GUS fusion |
| 171Bpro-R-SmaI | TCCCCCGGGTAAAACCACTCTTGTTCGACTAT | promoter GUS fusion |
| 171Cpro-F-PstI | CCATTTGTCCTGCTGCAGAACCG | promoter GUS fusion |
| 171Cpro-R-SmaI | TCCCCCGGGTCGACTCTTCAGTTGCTTATTAC | promoter GUS fusion |
| TRY-F-KpnI | GGGGTACCATGGATAACACTGACCGTCG | Over-expression |
| TRY-R-PstI | AACTGCAGCTAGGAAGGATAGATAGAAAAG | Over-expression |
| TCL1-F-KpnI | GGGGTACCATGGATAACACAAACCGTCTTC | Over-expression |
| TCL1-R-PstI | AACTGCAGTCATTTGTGGGAGAAATAGTC | Over-expression |
| MIR171A-F-KpnI | GGGGTACCCAAAGCATCTTCATCGATGTTC | Over-expression |
| MIR171A-R-PstI | AACTGCAGCAAGCACGAATTGCTTTACATTG | Over-expression |
| MIR171B-F- KpnI | GGGGTACCAACTAAATTTGTTAGGCCACTG | Over-expression |
| MIR171B-R-PstI | AACTGCAGCAAACATACTTCCGGAAATGTGG | Over-expression |
| MIR171C-F-KpnI | GGGGTACCATTTTGTTTGATATTTATTATTTC | Over-expression |
| MIR171C-R-BamHI | CGGGATCCTAATTAAAAGTTCAGAATATACAC | Over-expression |
| Chip-A1-F | GTTGTCTTGTAAGGCAAAAC | ChIP, region A1 |
| Chip-A1-R | TATCGCCAAACAGATAAAAC | ChIP, region A1 |
| Chip-A2-F | GTCACTGTTGTTGGTATAAG | ChIP, region A2 |
| Chip-A2-R | CATATTCATTCATCATCAAC | ChIP, region A2 |
| Chip-A3-F | CTTCTGCGAGATTCCCTCAA | ChIP, region A3 |
| Chip-A3-R | TTTCCTTCTTGAGGAGGTTC | ChIP, region A3 |
| Chip-A4-F | AGACAGAGGAGGAGACAAAG | ChIP, region A4 |
| Chip-A4-R | CTACCAAAGCACAAAAGCTG | ChIP, region A4 |
| Chip-B1-F | TGATAGGAACTTAACAAGAG | ChIP, region B1 |
| Chip-B1-R | TGCTAGATTGCTAGATAACG | ChIP, region B1 |
| Chip-B2-F | GGTCTAATTTAATGCTTGTC | ChIP, region B2 |
| Chip-B2-R | AGGGACGTCAGAAACCAAAG | ChIP, region B2 |
| Chip-B3-F | TCTTCTAATTCGTACTTTGG | ChIP, region B3 |
| Chip-B3-R | TAAAACCACTCTTGTTCGAC | ChIP, region B3 |
| Chip-C1-F | TTATTTTTATTGGTTGATGC | ChIP, region C1 |
| Chip-C1-R | AGTACCAAACCGACCCGAAC | ChIP, region C1 |
| Chip-C2-F | CGGACGACCTAAATATTTTG | ChIP, region C2 |
| Chip-C2-R | GGGTTTGCAGAGTGAAGGAG | ChIP, region C2 |
| Chip-C3-F | CATTGAAATAGCTCATGTTG | ChIP, region C3 |
| Chip-C3-R | ACTCTTCAGTTGCTTATTAC | ChIP, region C3 |
| TUB2-Chip-S | TAAATTCTGAACCCATTGTTTCTCA | ChIP, region control |
| TUB2-Chip-AS | AGTCCGATGATTGGCTTTATTATTC | ChIP, region control |
| miR171 probe | CGTGATATTGGCACGGCTCAATCA | RNA blots |
| miR156 probe | GTGCTCACTCTCTTCTGTCA | RNA blots |
